# Adaptive mechanisms and genomic plasticity for drought tolerance identified in European black poplar (*Populus nigra* L.)

**DOI:** 10.1093/treephys/tpw017

**Published:** 2016-08-01

**Authors:** Maud Viger, Hazel K. Smith, David Cohen, Jennifer Dewoody, Harriet Trewin, Marijke Steenackers, Catherine Bastien, Gail Taylor

**Affiliations:** 1 Centre for Biological Sciences, University of Southampton, Life Sciences Building, Southampton SO17 1BJ, UK; 2 UMR Ecologie et Ecophysiologie Forestières, INRA NANCY-Lorraine, 54280 Champenoux, France; 3 UMR Ecologie et Ecophysiologie Forestière, Université de Lorraine, BP 239, F-54506 Vandoeuvre, France; 4 Ministry of the Flemish Community, Research Institute for Nature and Forest (INBO), Geraardsbergen B-9500, Belgium; 5 INRA, Unité de Recherche Amélioration Génétique et Physiologie Forestières, 2163 avenue de la Pomme de Pin, CS 40001 Ardon, 45075 Orléans Cedex 2, France; 6 Present address: USDA Forest Service, National Forest Genetics Lab, 2480 Carson Road, Placerville, CA 95667, USA

**Keywords:** carbon isotope discrimination (Δ^13^C), microarray, stomatal number, water deficit

## Abstract

Summer droughts are likely to increase in frequency and intensity across Europe, yet long-lived trees may have a limited ability to tolerate drought. It is therefore critical that we improve our understanding of phenotypic plasticity to drought in natural populations for ecologically and economically important trees such as *Populus nigra* L. A common garden experiment was conducted using ∼500 wild *P. nigra* trees, collected from 11 river populations across Europe. Phenotypic variation was found across the collection, with southern genotypes from Spain and France characterized by small leaves and limited biomass production. To examine the relationship between phenotypic variation and drought tolerance, six genotypes with contrasting leaf morphologies were subjected to a water deficit experiment. ‘North eastern’ genotypes were collected at wet sites and responded to water deficit with reduced biomass growth, slow stomatal closure and reduced water use efficiency (WUE) assessed by Δ^13^C. In contrast, ‘southern’ genotypes originating from arid sites showed rapid stomatal closure, improved WUE and limited leaf loss. Transcriptome analyses of a genotype from Spain (Sp2, originating from an arid site) and another from northern Italy (Ita, originating from a wet site) revealed dramatic differences in gene expression response to water deficit. Transcripts controlling leaf development and stomatal patterning, including *SPCH*, *ANT*, *ER*, *AS1*, *AS2*, *PHB*, *CLV1*, *ERL1–3* and *TMM*, were down-regulated in Ita but not in Sp2 in response to drought.

## Introduction

Forests in Europe and elsewhere are likely to experience unprecedented rises in temperature and increases in the frequency and intensity of summer droughts in the future (Lindner et al. 2010, IPCC 2014). The capacity for long-lived forest trees to adapt to a changing climate is determined by adjustments to morphological and physiological functional traits. This phenotypic plasticity allows trees to respond to a rapidly changing climate and thus provides a mechanism for acclimation (Bussotti et al. 2015). Although recent droughts in Europe have had major effects on forest tree mortality (Solberg 2004, Bréda and Badeau 2008, Allen et al. 2010), high phenotypic plasticity could enable populations to survive in a changing environment (Benito Garzón et al. 2011), where moderate droughts will be increasingly common. As such, understanding phenotypic responses to drought provides an important insight into likely long-term genetic adaptations (Alberto et al. 2013).

The physiological responses to drought are complex and traits vary in their importance depending on severity, duration and timing of the drought (Bréda and Badeau 2008, Tardieu and Tuberosa 2010). These traits present as reduced leaf size and number, abscisic acid (ABA)-dependent and -independent signalling, lowered stomatal aperture and numbers, reduced stomatal conductance (*g*_s_), decreased leaf growth, altered patterns of root development and improved water use efficiency (WUE) (Tardieu and Tuberosa 2010). Moreover, microarray studies on linkage between physiological responses and underlying regulatory genes as well as metabolic networks in response to drought are elucidated in model plant species, including poplar (*Populus deltoides* Marshall and *Populus trichocarpa* T. & G. (Street et al. 2006), *Populus euphratica* Olivier (Bogeat-Triboulot et al. 2007), *P. deltoides* × *Populus nigra* L. (Cohen et al. 2010), *Populus balsamifera* L (Hamanishi et al. 2010, 2015) and *P. nigra* × *Populus maximowiczii* A. Henry (Wilkins et al. 2009)).

Furthermore, microarray studies on drought tolerance in *P. balsamifera* have identified variation in the pattern of transcript abundance between genotypes, which was correlated to growth maintenance after a water deficit (Hamanishi et al. 2010). These important studies generally focus on using commercial tree genotypes to elucidate gene expression changes that may be involved in determining water deficit responses. Although *Populus* is often defined as sensitive to drought, large variations in traits related to drought tolerance and water stress response have been reported, but generally in *F*_1_ or *F*_2_ hybrids of commercial value, and not for a wild collection such as described here. For example, osmotic adjustment varies across *F*_1_ and *F*_2_ genotypes (Marron et al. 2002, Tschaplinski et al. 2006), as does leaf expansion (Rae et al. 2009), leaf abscission (Street et al. 2006), WUE (Rae et al. 2004, Monclus et al. 2005, 2006, Voltas et al. 2006, Dillen et al. 2008) and Δ^13^C (Monclus et al. 2012). Stomatal traits linked to improved drought tolerance are complex and related to both stomatal function (opening and closing) and stomatal development and patterning. However, there is a limited understanding of genomic responses to drought in wild collections collected across a large geographical scale, which may harbour genetic potential for adaptation and increasingly provide the focus for broad geographical spanning genomic and genetic analysis of links between traits and genes. Recently, the potential to exploit natural genetic variation has been recognized in *Arabidopsis* with genome-wide association studies for traits becoming routine (Atwell et al. 2010), but the relevance of *Arabidopsis* for understanding tree adaptation may be limited (Taylor 2002). Drought tolerance is an obviously multigenic trait and genomic technologies allow the investigation of such traits, in contrast to traditional single gene studies that can limit the focus to the interaction between a small number of genes and, therefore, impede the identification of different pathways involved in drought response and adaptation.

European black poplar (*P. nigra*) is a riparian species that is widely distributed in Europe, North Africa and Central and West Asia (Vanden Broeck 2003). It has many economic uses, including domestic plantations and breeding programmes (Vanden Broeck 2003). Ecologically, *P. nigra* is a keystone riparian species (Vanden Broeck 2003), threatened by river drainage, water management (Gaudet et al. 2008) and climate change. Understanding phenotypic plasticity of *P. nigra* in response to drought is important. *Populus* is also widely accepted to be a model tree since it is fast growing, its genome is fully sequenced and there are a wide array of applicable genomic and genetic resources available (Taylor 2002, Tuskan et al. 2004, 2006, Jansson and Douglas 2007). Although poplars are considered sensitive to drought as they are abundant in riparian environments and often have a high demand for water (Dreyer et al. 2004, Street et al. 2006), considerable variation in response to water deficit has been observed between genotypes of *Populus* (Marron et al. 2002, Monclus et al. 2006, Street et al. 2006, Huang et al. 2009, Regier et al. 2009, Cocozza et al. 2010, Viger et al. 2013).

The aims of this study were (i) to quantify natural variation of productivity and other water use-associated traits in a broad, natural collection of black poplar, and examine the relationship between these traits and tree adaptation and their region of origin, which differ particularly in precipitation, (ii) to quantify phenotypic plasticity in response to drought in a group of genotypes and (iii) to determine the transcriptomic differences underlying drought tolerance in extreme genotypes from this natural collection.

## Materials and methods

### Common garden experiment

#### Plant material and growth conditions

**Figure 1.**
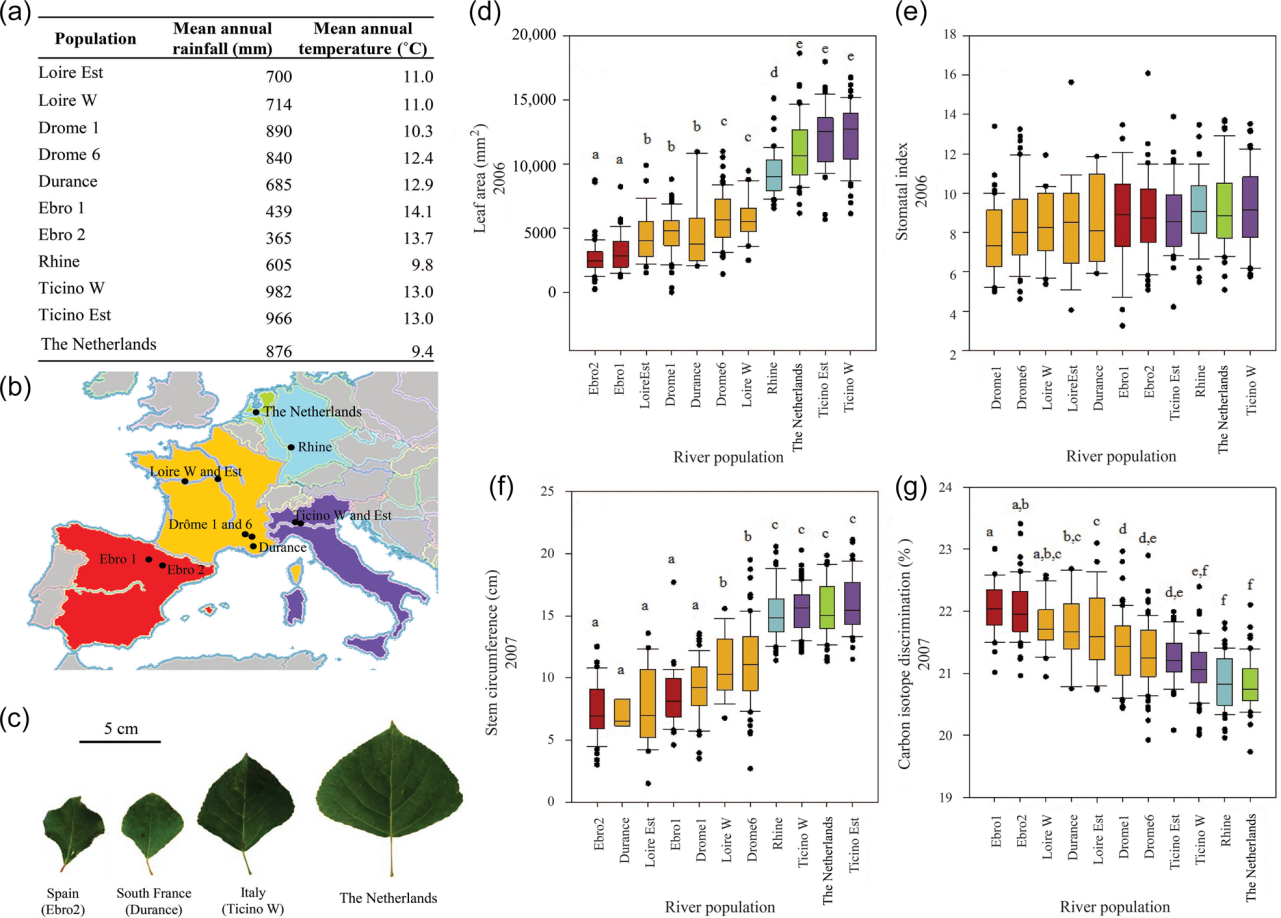
Association population information and measurements from the common garden experiment in Belgium: mean annual rainfall and temperature per river population (a), map of the 11 river populations of *P. nigra* collected in five European countries (b), leaf size and shape variation between populations (c), leaf area in mm^2^ (d), SI in % (e), stem circumference in cm (f) and wood carbon isotope discrimination in ‰ (g). Same letter indicates no significant difference at the 5% level, Student–Newman–Keuls post hoc testing. Each value with bars represents the average ± standard error.

Cuttings of 479 genotypes of *P. nigra* from wild populations were collected from five different European countries, including Spain, France, Italy, Germany and The Netherlands (see Table S1 available as Supplementary Data at *Tree Physiology* Online). Genotypes were grouped into 11 populations related to the river system near the collection (Figure 1). Hardwood cuttings were planted in a common garden in Belgium, Geraardsbergen (50°46′24″N, 3°52′56″E) in spring 2004, cut at the base in the spring of 2005 and side stems removed so that trees grew as single stems in June 2005. The experiment followed a randomized block design with six blocks each containing one replicate of each genotype with a double row of the commercial *Populus* genotype ‘Muur’ planted around the six blocks to minimize edge effects. The trees were planted at 0.75 × 2 m spacing. The site was rain-fed and not fertilized between March and September, but it was weed controlled and treated with fungicides every 3 weeks during these months in 2004–07.

#### Assessing phenotypic traits in the *P. nigra* collection

Each replicate was assessed for 12 morphological traits over three growing seasons (2005, 2006 and 2007). The youngest fully mature leaf was harvested, traced while fresh and placed in a paper bag. Leaf outlines were scanned using an Umax Astra 6700 scanner and assessed using ImageJ software (Image J.1.32.j, NIH, Bethesda, MD, USA). Leaf outlines were used for the measurement of leaf area, leaf length and leaf width, and calculating leaf ratio (length : width). Leaves collected in the second growing season (2005) were placed in paper bags then dried for 48 h at 80 °C, and weighed to calculate specific leaf area (SLA) as the ratio of leaf area to leaf dry weight.

Epidermal cell number and size were measured using cell imprints taken in 2006 from the first interveinal region of the abaxial surface of the first fully mature leaf following the methods of Gardner et al. (1995). Images of cell imprints were assessed in ImageJ (Abràmoff et al. 2004) to count the number of cells and stomata per unit area and average cell area of 10 cells per leaf. Subsequently, stomatal density (ratio of stomata number per unit area), stomatal index (SI, ratio of the number of stomata per total cell number as a percentage) and cell number per leaf, estimated as the ratio of leaf area to cell area, were calculated. Height was recorded following the first year of growth (2005), and circumference 1 m above ground level was assessed following the second (2006) and third year of growth (2007).

Improved WUE is also associated with severe drought, where WUE is the ratio between net carbon assimilation (*A*) and transpiration rate (*E*), and is negatively associated with carbon isotope discrimination (Δ^13^C) or positively correlated with carbon isotope composition (δ^13^C) (Farquhar and Richard 1984, Farquhar et al. 1989, Condon et al. 2002). Wood was collected for Δ^13^C measurement in March 2007, with 30 cm sections cut from 40 cm above ground. These samples were stored in a cold room in individual plastic bags before being debarked and cut into small pieces. Samples were dried in the oven for 48 h at 80 °C before being ground using a ball grinder (Glen Creston ball, Retsch MM300, London, UK) and stored in a glass container. One milligram of material was weighed and placed into a 6 × 4 mm tin capsule (Elemental Microanalysis, Devon, UK). Samples were analysed using a SerCon 20-20 Stable Isotope Analyser with ANCA-GSL Solid/Liquid Preparation Module (SerCon, Crewe, UK). Carbon isotope composition was determined by δ^13^C (‰) = δ_plant_ = [(*R*_sample_ − *R*_reference_)/*R*_reference_] × 1000, where *R*_sample_ and *R*_reference_ are the ^13^C/^12^C ratios of the sample and the reference, respectively, in Vienna Pee Dee Belemnite units (Scrimgeour et al. 2004). Carbon isotope discrimination was calculated as Δ^13^C (‰) = [(δ_air_ − δ_plant_)/(1 + (δ_plant_/1000)] with δ_air_ assumed to be −8‰ (Farquhar and Richard 1984, Monclus et al. 2006).

### Drought experiment

#### Plant material and growth conditions

In order to examine phenotypic plasticity related to water deficit, a subset of trees was chosen for a moderate drought glasshouse experiment in Southampton, UK. Six genotypes were selected from the *P. nigra* collection (see Table S2 available as Supplementary Data at *Tree Physiology* Online): four from the extreme ‘leaf size’ genotypes (two Spanish ‘small leaf’, Sp1, Sp2; one Italian ‘large leaf’, Ita; and one from the Netherlands ‘large leaf’, NL) and two from the Drôme population in France (Fr1 and Fr2). These genotypes were chosen as typical ‘small-leaf’ genotypes from arid areas. The French genotypes were selected to represent a typical leaf morphology from a broad range in temperature and precipitation patterns. Cuttings were planted in John Innes No. 2 (John Innes, Norwich, UK) without fertilization in January 2007 in a glasshouse and cut back in November 2007 at 10 cm from the base. From November 2007, the trees were watered daily and put into dormancy conditions (natural light, 15 °C : 13 °C, day : night). In May 2008, the temperature in the glasshouse was set at 22 °C : 16 °C, day : night. During the experiment, photoperiod was maintained 16 h : 8 h, light : dark with a minimum photosynthetic active radiation at the top of the plants of 150 μmol m^−2^ s^−1^, supplementing natural sunlight. The number of replicates for each genotype varied between 5 and 10 plants per condition (see Table S2 available as Supplementary Data at *Tree Physiology* Online). The trees were randomized in 10 blocks containing one replicate per genotype per treatment.

At the beginning of the experiment on 1 September 2008, 200 ml of water was added to each potted tree and the pots were then covered in aluminium foil to prevent water evaporation. The first mature leaf and the first emerging young leaf were tagged with cotton string. From 1 September until 1 October, soil moisture content was measured every morning with a Delta-T ML2x ThetaProbe connected to an HH2 moisture meter (Delta-T Devices, Cambridge, UK). Well-watered trees (control) were watered to field capacity and drought-stressed trees were kept between 15 and 20% volume soil moisture as has been determined as a suitable moderate drought treatment for poplar (Street et al. 2006). Using a repeated measurements test over time, soil moisture content showed significant differences between treatment (*F*_1,50_ = 363.17, *P* < 0.001) but no significant differences between genotypes (*F*_5,50_ = 1.06, *P* = 0.392) and no genotype × treatment interaction effect (*F*_5,50_ = 0.82, *P* = 0.543), meaning all the genotypes had their soil moisture decreased equally under drought (see Figure 3, see Figures S1 and S2 available as Supplementary Data at *Tree Physiology* Online).

#### Physiological and growth measurements

Biomass measurements were conducted on 1 September 2008 (0 day after drought (DAD)) and 17 September 2008 (16DAD). Measures included height, stem diameter measured using digital callipers at 10 cm from the stem base, the number of branches and the number of leaves. Growth was calculated as the difference between 0DAD and 16DAD for stem height and diameter, as well as number of branches and leaves. Leaves newly developed (NLN) during the experiment above the tag on the first emerging leaf were also counted at 16DAD and used with the total number of leaves at 0DAD and 16DAD to calculate the number of fallen leaves, as senescence = (NL_16DAD_ − NL_0DAD_) − NLN. The third mature leaf (counting from the uppermost mature leaf) was sampled at 27DAD, traced and dried as described above. Dried leaves were used to calculate SLA, the ratio of leaf area (prior to drying) to leaf dry mass (Marron et al. 2005).

The first three leaves that emerged on the main stem during the experiment were followed for leaf area using the leaf tagged on 0DAD. The contour of the leaves was traced onto paper before the images were scanned and processed using ImageJ (Abràmoff et al. 2004). Stomatal conductance was measured on the first mature leaf tagged at 0DAD, 5DAD and 15DAD, using a steady-state porometer (LI-1600; LI-COR, Inc., Lincoln, NE, USA). In order to examine variation in WUE, a young leaf (third leaf from the top) of each tree was placed in a paper bag on 19DAD and oven dried. Δ^13^C was measured as described for the wood collected in Belgium.

#### Gene expression analysis

Young leaves were sampled on 19DAD for gene expression analyses (microarrays and real-time polymerase chain reaction (PCR)). Two genotypes—one from Spain (Sp2) and one from Italy (Ita)—were selected for microarray analysis based on being the most extreme genotypes in terms of morphology. Each sample (the first two unfurled leaves) was flash frozen in liquid nitrogen and stored at −80 °C for further analysis. RNA was extracted following the cetyl trimethlammonium bromide protocol from Chang et al. (1993). Eight RNA samples, corresponding to two biological replicates of both well-watered and drought treatments per genotype, were sent to the European Arabidopsis Stock Centre (NASC, Loughborough, UK) microarray service for the cDNA synthesis, fragmentation, array hybridization and scanning using Affymetrix GeneChip Poplar Genome Arrays (Affymetrix, Santa Clara, CA, USA). Affymetrix CEL files were imported into R software (R Development Core Team 2014). Probe sets exhibiting no signal intensity were filtered out by a Present call procedure as described by McClintock and Edenberg (2006). Briefly, CEL files were normalized using the MAS5 algorithm with default parameters (affy package, v1.48.0). MAS5 provides a detection call, Absent (A), Present (P) or Marginal (M), which indicates whether the specific transcript is detectable. For each probe set, the percentage of Present calls in each condition was calculated. Probe sets that exhibited a percentage of Present calls of 100% in at least one condition for both genotypes were kept. The other probe sets were removed from the analysis. This procedure also allowed probe sets that hybridized exclusively to one genotype to be discarded (Cohen et al. 2010). Finally, 31,084 validated probe sets were retained. In order to compute differential gene expression, CEL files were then normalized using the RMA algorithm with default parameters (affy package, v1.48.0). Differential expression was calculated as log_2_(fold change) between drought and control samples for the 31,084 validated probe sets. Statistical significance of differential expression was tested using moderated *t*-tests implemented in the eBayes function (limma package v3.24.12, Smyth 2004) and false discovery rate (FDR) corrections for multiple testing were applied. Thresholds of |log_2_(FC)| ≥ 1 and corrected *P* < 0.05 were used to identify differentially expressed genes. Probe sets were annotated using the Poparray website (http://aspendb.uga.edu/poparray) and assigned to a *Populus* gene model (v. 3.0) and its closest *Arabidopsis* homologue, and gene ontology (GO) biological process, cellular component and molecular function classifications.

The software MapMan (Thimm et al. 2004) was used for pathway analysis. Statistics (Wilcoxon Rank Sum Test with a Benjamini–Hochberg FDR correction) were implemented in MapMan to reveal BINs (groups of functionally similar items—genes, enzyme activities, metabolites, used to construct pathways) exhibiting a significant difference in expression profile behaviour compared with the other BINs. Gene ontology enrichment was also studied using the parametric analysis of gene set enrichment (PAGE) tool on the AgriGo website (Du et al. 2010) with default parameters using validated probe sets as the reference.

Results of the microarray experiment were confirmed using quantitative real-time PCR (qPCR) for a set of differentially expressed candidate genes. Forward and reverse primers were designed, from the *P. trichocarpa* genome (v1.2), specifically to each gene (see Table S3 available as Supplementary Data at *Tree Physiology* Online). Reverse transcription of RNA to cDNA was performed using the ImProm-II Reverse Transcription kit (Promega UK, Southampton, UK) following the manufacturer’s instructions. Each qPCR was composed of 5 µL 2× Precision-SY Master Mix (PrimerDesign Ltd, Southampton, UK), 5 pmol forward and reverse primers and 25 ng diluted cDNA. Plates were run on a Chrom4 Real-Time PCR Detection System (Bio-Rad Laboratories, Hercules, CA, USA). Reactions were incubated at 95 °C for 10 min and then 40 cycles of 15 s at 95 °C, 1 min at 60 °C and a plate read, followed by an incubation at 72 °C for 10 min. A melting curve was then performed from 60 to 95 °C with a read every 0.2 °C and 1 s hold, in order to check for primer dimers, DNA contamination and secondary products. Values were exported with the software Opticon Monitor 3.1 (Bio-Rad Laboratories). Amplification efficiency was measured following the equation from Liu and Saint (2002):

$$E={\left(\frac{R_{n,A}}{R_{n,B}}\right)}^{\left[1/(C_{T,A}-C_{T,B})\right]}-1$$

where *R*_n,A_ and *R*_n,B_ are *R*_n_ at arbitrary thresholds A and B in an individual curve, respectively, and *C*_T,A_ and *C*_T,B_ are the threshold cycles at these arbitrary thresholds (Liu and Saint 2002).

Ratios were calculated as:

$$\frac{E\;(\mathrm{control}-\mathrm{drought})\;\mathrm{target}}{E\;(\mathrm{control}-\mathrm{drought})\;\mathrm{reference}}$$

#### Statistical analysis

Data from the Belgium common garden experiment were analysed using the SPSS software package (SPSS, Chicago, IL, USA). Kolmogorov–Smirnov tests were used to test for normality, and transformation (log_10_) was carried out when required. A general linear model (GLM) tested the effects of block and river population:

$$Y_{ij}=\mu +\alpha_{i}+\beta_{j}+\epsilon$$

where μ is the mean, *Y_ij_* is the phenotype in the *i*th block and in the *j*th river population, α*_i_* is the block effect, β*_j_* is the river population effect and ϵ is the residual error. A comparison of means was carried out between river populations using a Student–Newman–Keuls post hoc test.

A GLM was also performed to test genotype effects:

$$Y_{i}=\mu +\alpha_{i}+\epsilon$$

where *Y_i_* is the phenotype in the *i*th genotype, α*_i_* is the genotype effect and ϵ is the residual error.

Climatic data from the region of origin for genotypes were correlated with and among phenotypic traits as measured in the common garden was tested using Spearman’s ρ in SPSS v19.0 (SPSS).

Data from the glasshouse experiment were also analysed for genotype and treatment effect (and their interaction) using the SPSS software package (SPSS). Normality (Kolmogorov–Smirnov test) and block effects were checked before performing an analysis of variance (ANOVA) GLM. Data were transformed using a natural log when required. A GLM tested the effects of genotype and treatment:

$$Y_{ij}=\mu +\alpha_{i}+\beta_{j}+\epsilon$$

where *Y_ij_* is the phenotype in the *i*th genotype and in the *j*th treatment, α*_i_* is the genotype effect, β*_j_* is the treatment effect and ϵ is the residual error. A comparison of means was carried out among genotypes using a Student–Newman–Keuls post hoc test. A test for repeated measurements was used for leaf area over time for each leaf number. Phenotypic plasticity in drought response was quantified using the equation [(drought−control)/control]×100 from Street et al. (2006).

## Results

### Common garden experiment

The collection of nearly 500 genotypes of *P. nigra* selected from contrasting climatic zones across Europe was used to study natural variation in wood carbon isotope discrimination (Δ^13^C), leaf, cell and biomass traits for trees grown under well-watered conditions in a Belgian common garden field site (Figure 1). Significant differences in plant morphology were observed between natural populations. Leaf area, stem circumference and Δ^13^C varied significantly between river sites (*F*_10,482_ = 129.8, *P* < 0.001; *F*_10,453_ = 35.2, *P* < 0.001; *F*_10,466_ = 33.5, *P* < 0.001, respectively, with Type III sums of squares). For SI, although the difference was not significant (*P* < 0.05), a trend was apparent with northern populations (Italy, The Netherlands and Germany) exhibiting a high SI, while genotypes from French populations showed a lower SI. In contrast to other measurements, the Spanish genotypes were intermediate in their ranking (Figure 1e). Leaf area and stem circumference were highest in the northern river populations (Italy, The Netherlands and Germany), while Spanish and southern French genotypes had the smallest leaves and stem circumference (Figure 1d and f). Δ^13^C tended to decrease along this north–south latitudinal gradient, although there were exceptions, such as for genotypes from west Loire (Figure 1g), but suggests that, under well-watered conditions, WUE was higher for genotypes from northern latitudes, when grown in the Belgian common garden.

**Figure 2.**
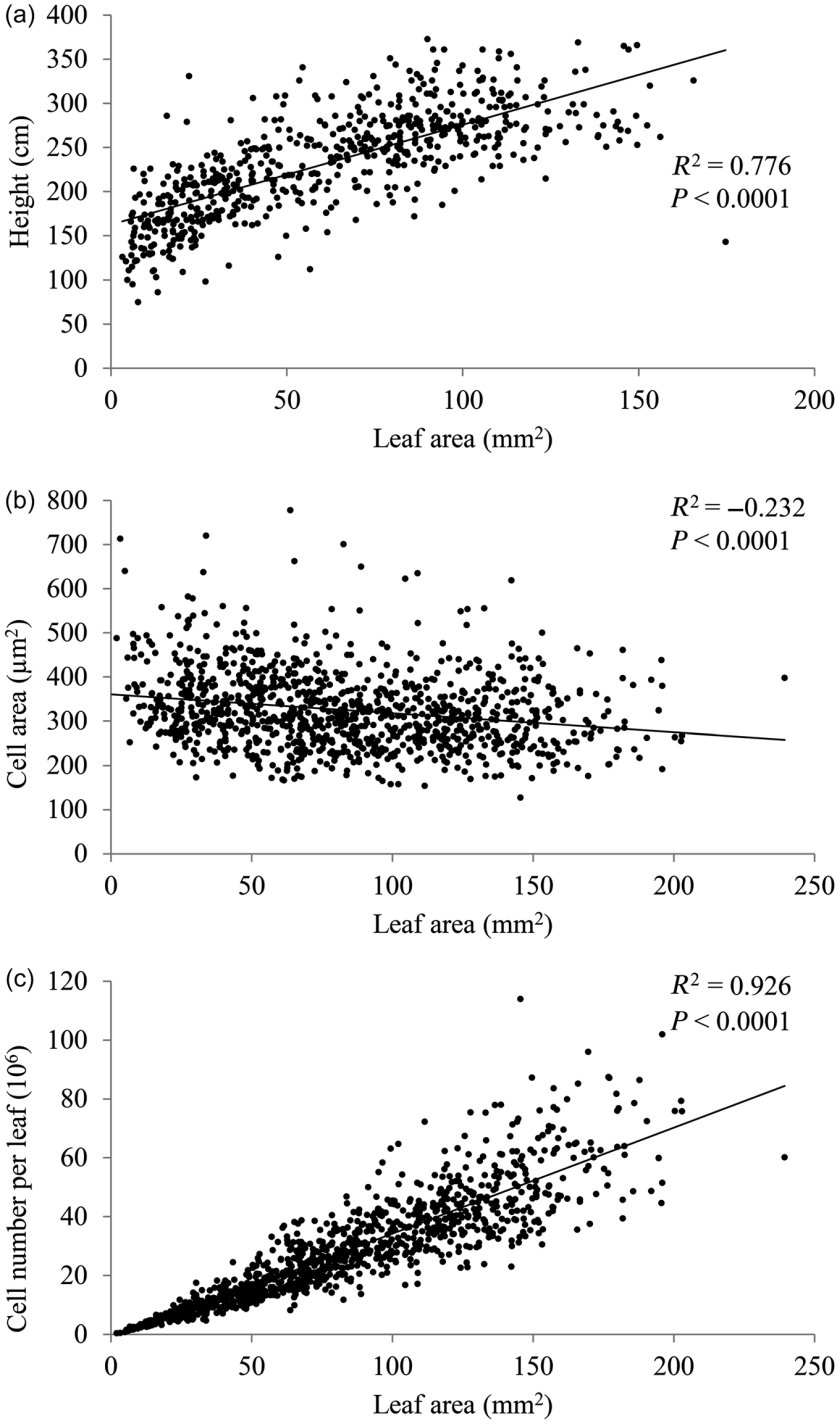
Correlations of biomass traits of interest: leaf area and height in 2005 (a), leaf area and cell area in 2006 (b) and leaf area and cell number per leaf in 2006 (c). Leaf traits are based on the youngest fully mature leaf from each tree. Spearman’s rho (*R*^2^_s_) and the probability that it differs from zero (*P*) are provided for each correlation.

Correlation between leaf, cell and biomass traits within and among growing seasons showed that leaf area correlated with tree height and circumference, both of which are woody biomass traits (see Table S4 available as Supplementary Data at *Tree Physiology* Online, Figure 2). In the third year of growth (2006), when leaf cell traits were measured, there was a strong positive correlation between cell number per leaf and leaf area (see Table S4 available as Supplementary Data at *Tree Physiology* Online, Figure 2, *R*^2^ = 0.927, *P *< 0.0001) but a weak negative correlation between cell area and leaf area (see Table S4 available as Supplementary Data at *Tree Physiology* Online, Figure 2, *R*^2^ = −0.235, *P* < 0.0001). Furthermore, stomatal patterning correlated strongly with all biomass traits with the exception of SI, which showed no relationship with leaf shape ratios in either 2005 or 2006 (*R*^2^ = −0.059, *P* < 0.85; *R*^2^ = 0.025, *P* < 0.429, respectively). Precipitation at the genotype site of origin correlated with leaf and stem phenotypic traits with higher precipitation (mean annual, minimum and maximum) correlated with increased leaf areas, which are made up of a greater number of smaller cells per leaf with more stomata, higher SLA and increased stem height and circumference (see Table S4 available as Supplementary Data at *Tree Physiology* Online). Additionally, higher temperatures (mean annual, minimum and maximum) correlated with leaf shape ratio and SLA. The temperature of the coolest month seems most important with respect to leaf area and cell number per leaf, as well as stem height and circumference in 2005 and 2006, respectively. However, mean annual temperature and the temperature of the warmest month also correlated with reduced leaf cell size and stem circumference and increased abaxial stomatal density (see Table S4 available as Supplementary Data at *Tree Physiology* Online).

### Drought experiment

**Figure 3.**
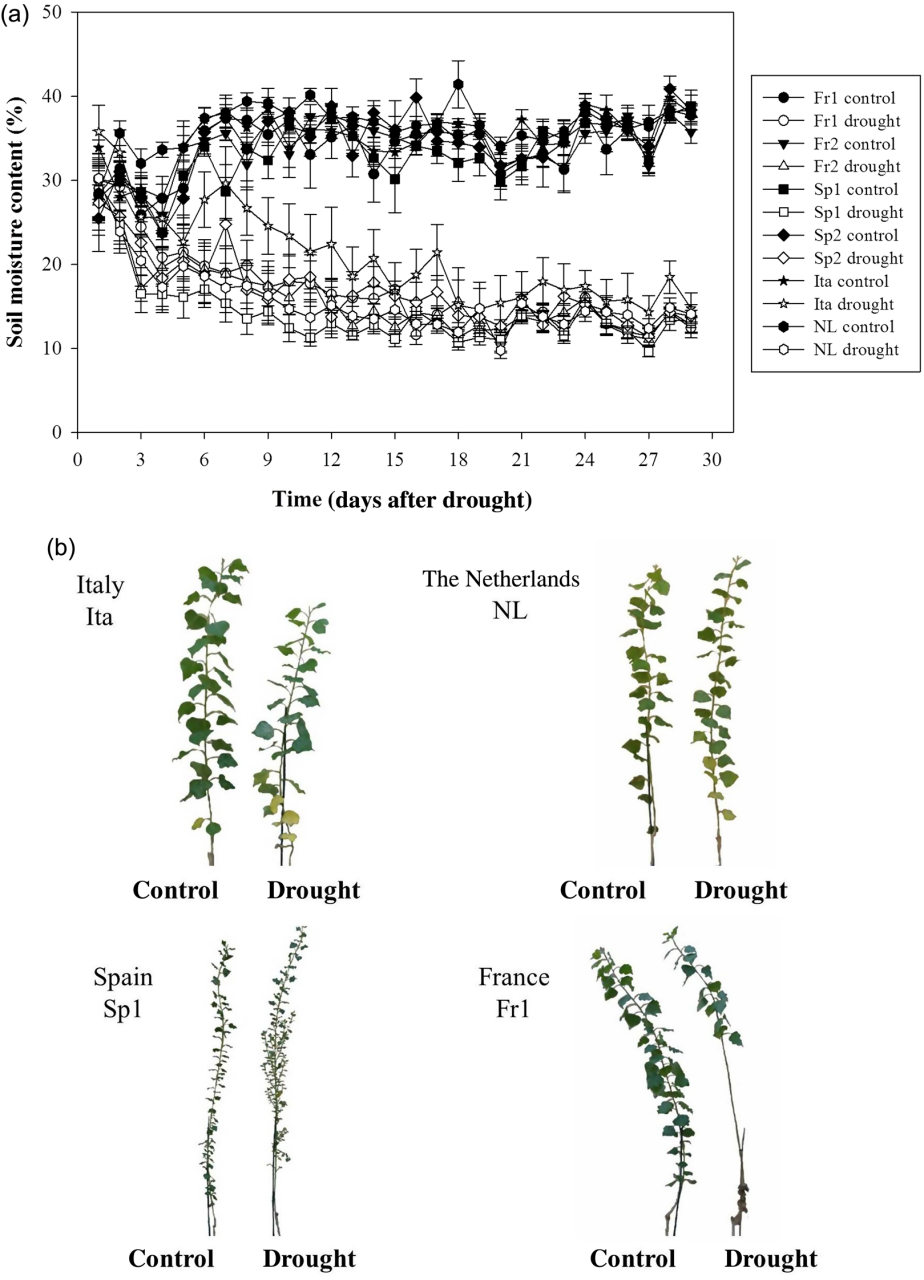
Soil moisture content (%) over time (days after drought) for each genotype (a). Filled symbols represent well-watered (control) and open symbols are for drought treatments. Each value with bars represents the average ± standard error. Photographic representation of the morphological effects of drought on the trees grown in the greenhouse (b).

Six contrasting genotypes were selected from the common garden trial to further elucidate phenotypic plasticity in response to drought, and how this varied across genotypes adapted to local drought conditions. These genotypes were subjected to a moderate drought in a controlled environment glasshouse in southern England (Figure 3a). Variation in response to drought was observed across the six selected genotypes (Table 1). Interaction between genotype and treatment was significant for Δ^13^C, from a two-way ANOVA, and close to significant (*P* < 0.10) for stem growth. Five of the nine traits measured showed both genotype and drought main ANOVA effects, while highly significant drought effects were observed for *g*_s_, Δ^13^C, leaf production and growth traits (Table 1). Furthermore, SLA varied significantly between genotypes but was unaffected by the drought treatment (Table 1).

**Table 1.** Summary of statistical results presenting the *F*-value and *P*-value for each trait using a GLM test for the main effects genotype and treatment and the interaction genotype × treatment. Bold values are significant (*P* < 0.05).

Trait	Genotype		Water treatment		Genotype × water treatment	
	*F*	*P*-value	*F*	*P*-value	*F*	*P*-value
*g*_s_ 5DAD	5.078	**<0.001**	15.860	**<0.001**	1.344	0.252
*g*_s_ 15DAD	1.469	0.207	103.092	**<0.001**	1.912	0.100
Δ^13^C	5.893	**<0.001**	7.511	**0.008**	2.567	**0.037**
Height growth	6.579	**<0.001**	37.086	**<0.001**	0.726	0.606
Stem diameter growth	2.116	0.071	14.77	**<0.001**	1.989	0.088
Branches formation	0.697	0.627	0.948	0.333	0.639	0.670
New leaf development	16.216	**<0.001**	24.964	**<0.001**	0.523	0.758
Leaf senescence	2.502	**0.036**	5.182	**0.025**	0.839	0.526
SLA	10.538	**<0.001**	2.977	0.088	0.923	0.470

#### Biomass

**Figure 4.**
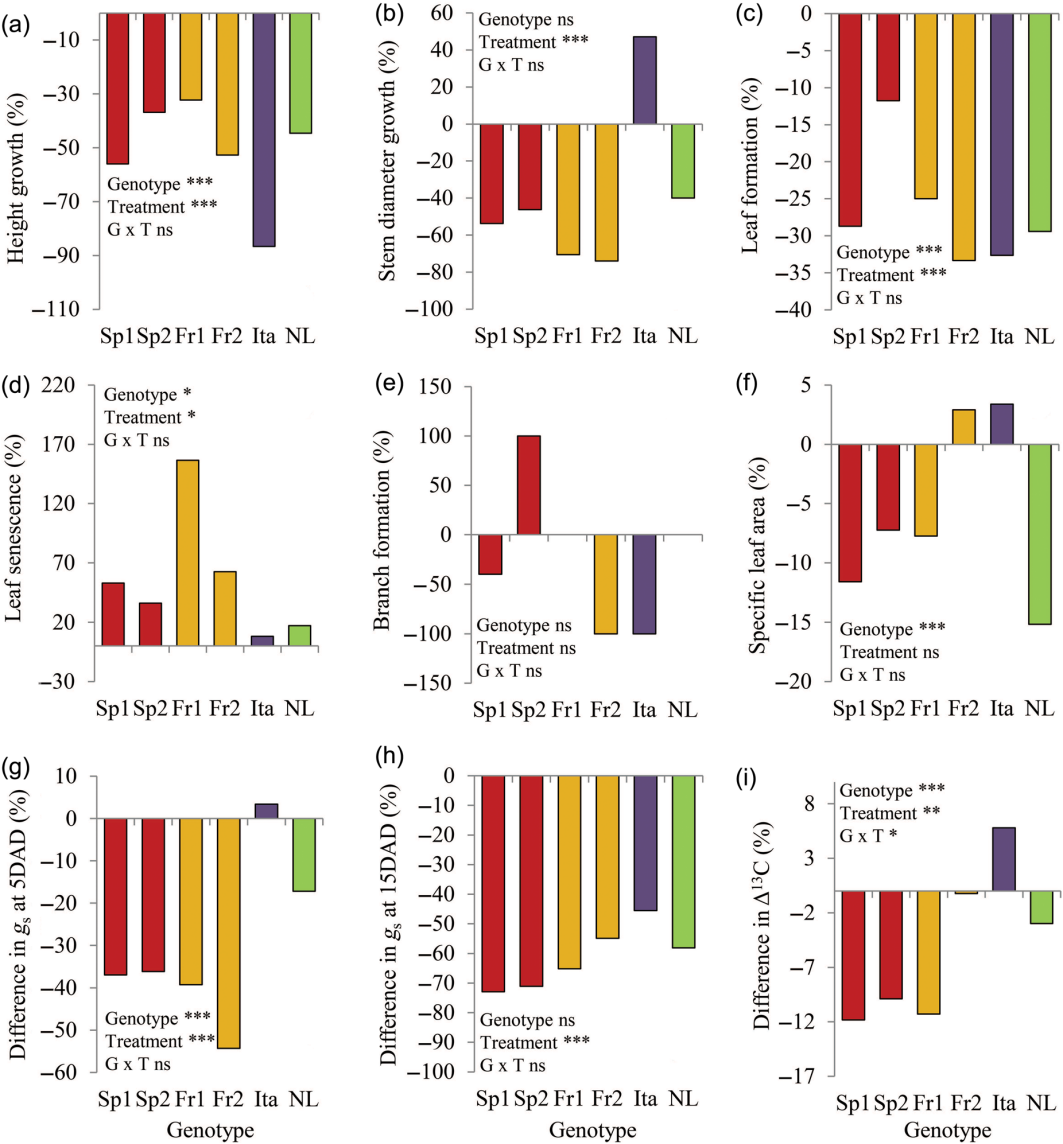
Percentage difference of biomass using the formula [(drought − control)/(control × 100)] from Street et al. (2006): height growth in mm (a), stem diameter growth in mm (b), new leaf formation (c), leaf senescence (d), branch formation (e), SLA in cm^2^ g^−1^ (f), *g*_s_ in μmol m^−2^ s^−1^ at 5 DAD (g) and 15 DAD (h), and carbon isotope discrimination in ‰ (i).

Images taken on 20DAD revealed the main morphological variation in response to drought across the six selected genotypes (represented by four genotypes in Figure 3b). Biomass production was also measured (see Table S5 available as Supplementary Data at *Tree Physiology* Online, Figure 4) and revealed that height growth decreased for all genotypes in response to drought (genotype: *F*_5,85_ = 6.6, *P* < 0.001; treatment: *F*_1,85_ = 37.1, *P* < 0.001) with the largest decrease (−86%) for the Ita genotype (Figure 4a). Fr1 and Sp2 maintained some height growth under drought with only moderate reductions apparent (−32 and −37%, respectively).

Differences between genotypes were apparent for both leaf production (formation) and leaf loss (senescence). Leaf production differed significantly between genotypes (*F*_5,91_ = 16.2, *P* < 0.001). In addition, leaf production was significantly affected by drought, particularly in Ita, Fr2 and NL (*F*_1,91_ = 25.0, *P* < 0.001, Figure 4c). One Spanish genotype (Sp2) continued to develop approximately the same number of new leaves during exposure to drought (an average of 6.0 leaves)—similar to that in well-watered conditions (6.8 leaves). In the well-watered treatment, Sp1 developed the most new leaves during the experiment (8.88), while trees from Italy only formed an average of 3.89 new leaves. Leaf senescence and loss on the main stem increased significantly under drought (*F*_1,86_ = 5.2, *P* = 0.025), but significant genotype effects were also apparent (*F*_5,86_ = 2.5, *P* = 0.036). French and Spanish genotypes lost more leaves (Figure 4d), while trees from Italy and the Netherlands largely retained leaves. Sp2 also developed two to four more branches on average in drought compared with well-watered conditions (Figure 4e). However, this trait did not show any significant genotype (*F*_5,81_ = 0.697, *P* = 0.627) or treatment (*F*_1,81_ = 0.948, *P* = 0.33) effects. Genotypes NL, Ita and Fr2 developed no branches in response to water deficit. Specific leaf area was measured at the end of the experiment and revealed a significant genotype (*F*_5,87_ = 10.5, *P* < 0.001) but not a treatment effect (*F*_1,87_ = 3.0, *P* = 0.09).

#### Stomatal conductance and carbon isotope discrimination

Stomatal conductance was measured during the progression of drought (see Table S5 available as Supplementary Data at *Tree Physiology* Online, Figure 4g and h). Early after the onset of drought (5DAD, Figure 4g), Spanish and French genotypes reacted quickly to water deficit with *g*_s_ declining rapidly by −54 and −36%, respectively (genotype: *F*_5,96_ = 5.1, *P* < 0.001, treatment: *F*_1,96_ = 15.9, *P* < 0.001). In contrast, the Ita genotype showed a small increase in *g*_s_ in response to drought (3.4%) and NL a moderate decline (−17%). After 15 days of drought (Figure 4h), these contrasting early responses of stomata to drought were no longer apparent and all genotypes showed a significant decline in *g*_s_ (*F*_1,92_ = 103.1, *P* < 0.001). Young leaves developed during the experiment were collected to measure Δ^13^C (Figure 4i). Δ^13^C showed significant variation between genotypes (*F*_5,58_ = 5.9, *P* < 0.001), a highly significant effect of drought (*F*_1,58_ = 7.5, *P* = 0.008) and a significant interaction of genotype × treatment (*F*_5,58_ = 2.6, *P* = 0.037), indicating that the response to drought differed depending on genotype. While Sp1, Sp2 and Fr1 decreased their Δ^13^C by ∼10% during the drought treatment, possibly indicating an increase in WUE, Fr2 showed no variation between treatment and Ita increased Δ^13^C under drought.

#### Leaf growth

**Figure 5.**
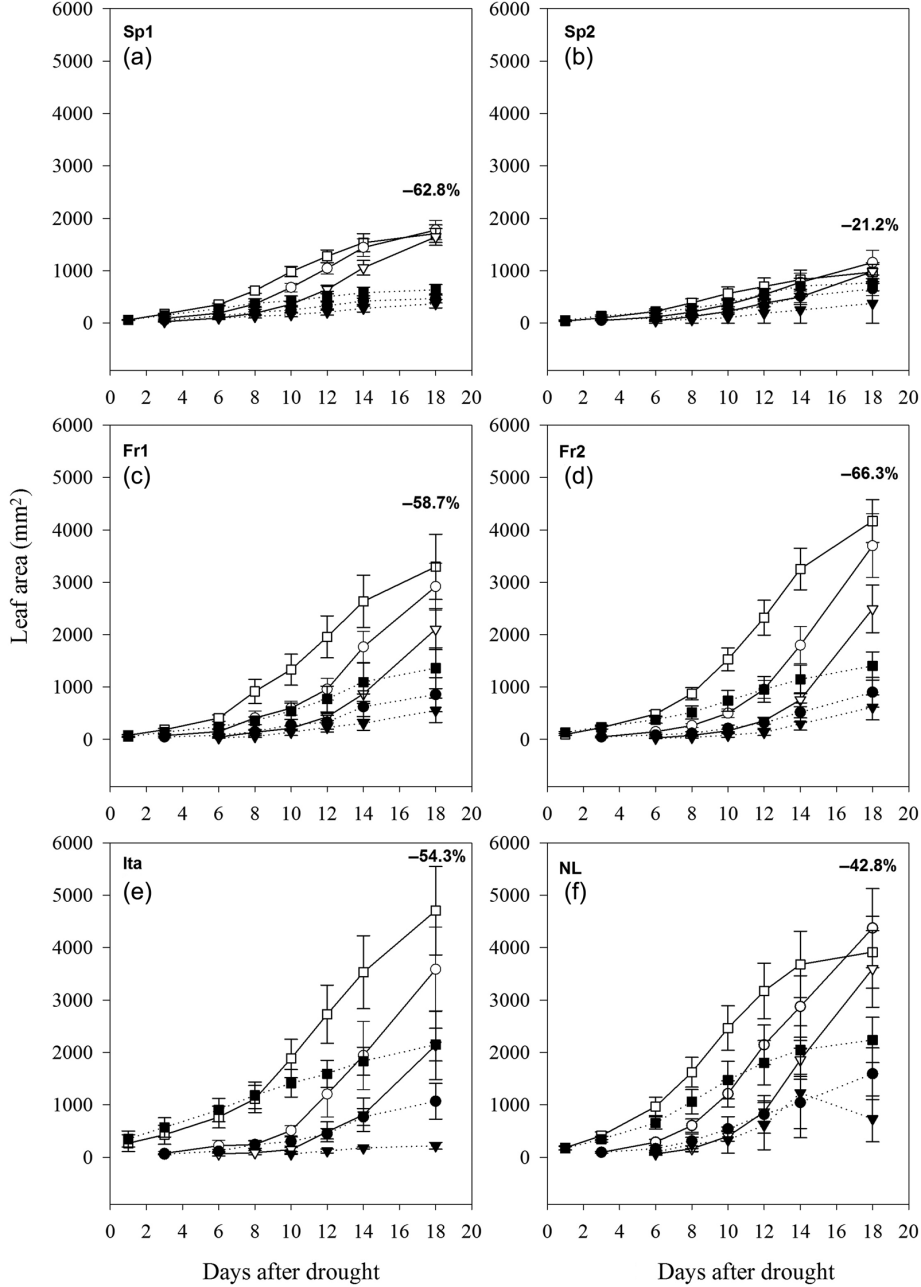
Leaf area development over time (days after drought) for the first emerging leaf (square), the second leaf emerging (circle) and the third leaf emerging (triangle) under well-watered conditions (solid lines and open symbols) and drought stress (broken lines and filled symbols) for each genotypes: Sp1 (a), Sp2 (b), Fr1 (c), Fr2 (d), Ita (e) and NL (f). Percentage difference in leaf area corresponds to the first emerging leaf after 18 DAD following the formula [(drought − control)/(control × 100)] from Street et al. (2006).

Leaf area was measured for the first three leaves emerging from 1 to 19DAD (Figure 5). Genotype had a significant effect on leaf area for all leaf numbers (Leaf 1: *F*_5,82_ = 7.538, *P* < 0.001; Leaf 2: *F*_5,54_ = 6.162, *P* < 0.001; Leaf 3: *F*_5,36_ = 6.328, *P* < 0.001). The effect of treatment was also significant (Leaf 1: *F*_1,82_ = 21.75, *P* < 0.001; Leaf 2: *F*_1,54_ = 26.86, *P* < 0.001; Leaf 3: *F*_1,36_ = 23.69, *P* < 0.001), but genotype and treatment did not interact. For the trees under well-watered conditions, both Spanish genotypes had the smallest leaves (1700 and 1000 mm^2^ on average, respectively) and the Italian had the largest leaves (4700 mm^2^ on average for Leaf 1). This rank order and size distribution was consistent with that observed in the common garden experiment, indicating that the greenhouse conditions did not change the phenotypic differences in these plants. Sp2 showed the smallest reduction in leaf area (−21.2%) and Fr2 the largest reduction (−66.3%) in response to drought.

#### Transcriptome response to drought

**Figure 6.**
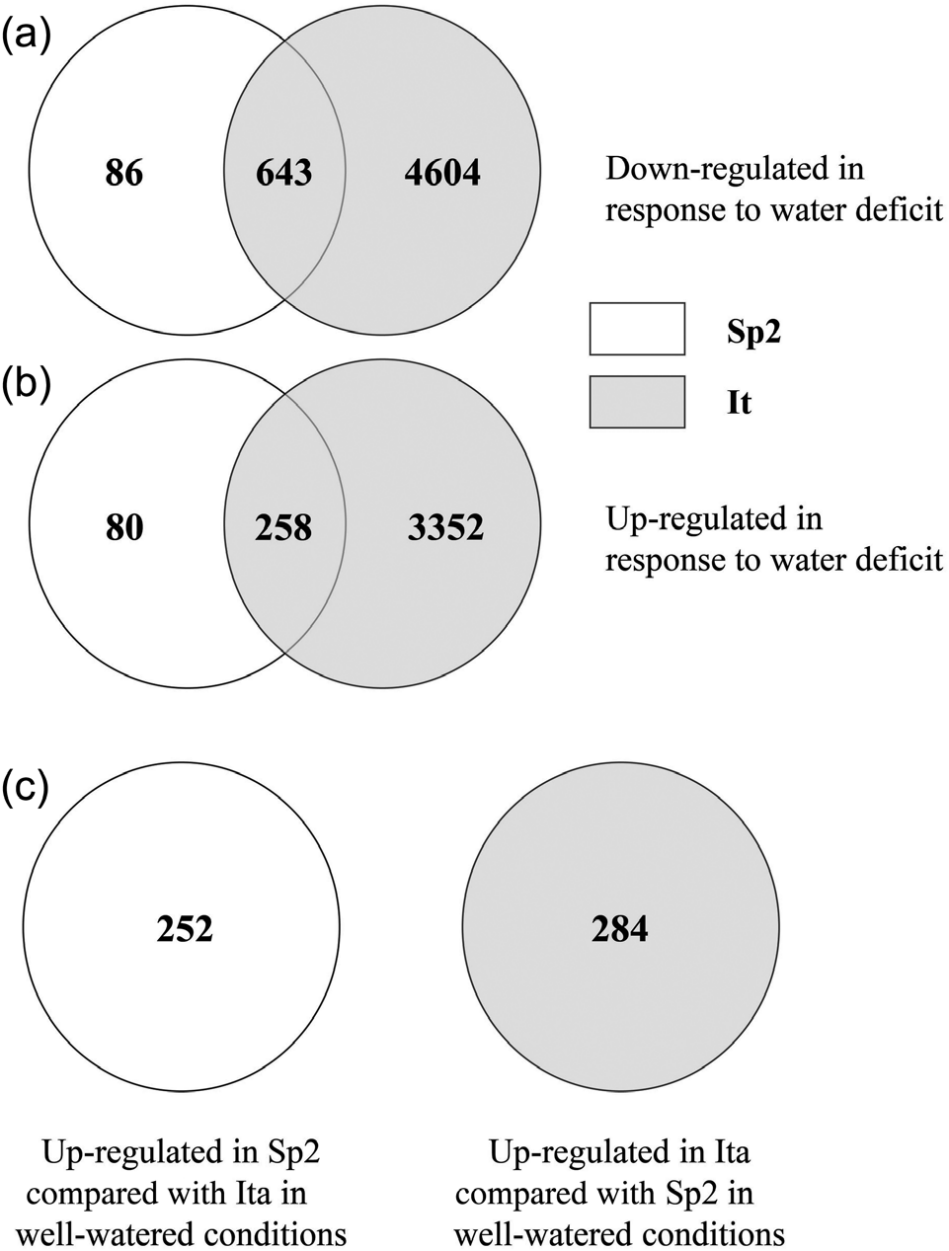
Venn diagram representing the Affymetrix ID probe sets that were twofold up-regulated (a) and down-regulated (b) in response to moderate drought—differentially expressed between the Spanish Sp2 (white) and the Italian Ita (grey) genotypes. Numbers in the circle overlap indicate the number of transcripts common to both genotypes and numbers outside the overlap indicate the number of transcripts exclusive to the genotype indicated. Circles (c) indicate the number of transcripts up-regulated and down-regulated in Sp2 compared with Ita in well-watered conditions.

Dramatic differences were apparent in the transcriptomic responses to drought in the contrasting Spanish and Italian genotypes selected for gene expression analysis (see Table S9 available as Supplementary Data at *Tree Physiology* Online). In the northern Italian genotype (Ita), 8857 probe sets displayed a significant twofold change in intensity in response to drought (3610 up-regulations and 5247 down-regulations, Figure 6a and b). In contrast, for the Spanish genotype (Sp2), only 1067 probe sets exhibited a twofold differential expression between control and drought conditions (338 up-regulations and 729 down-regulations, Figure 6a and b). Only 258 probe sets were commonly up-regulated between the two genotypes under drought and 643 were commonly down-regulated in response to drought (Figure 6c, see Table S9 available as Supplementary Data at *Tree Physiology* Online).

**Figure 7.**
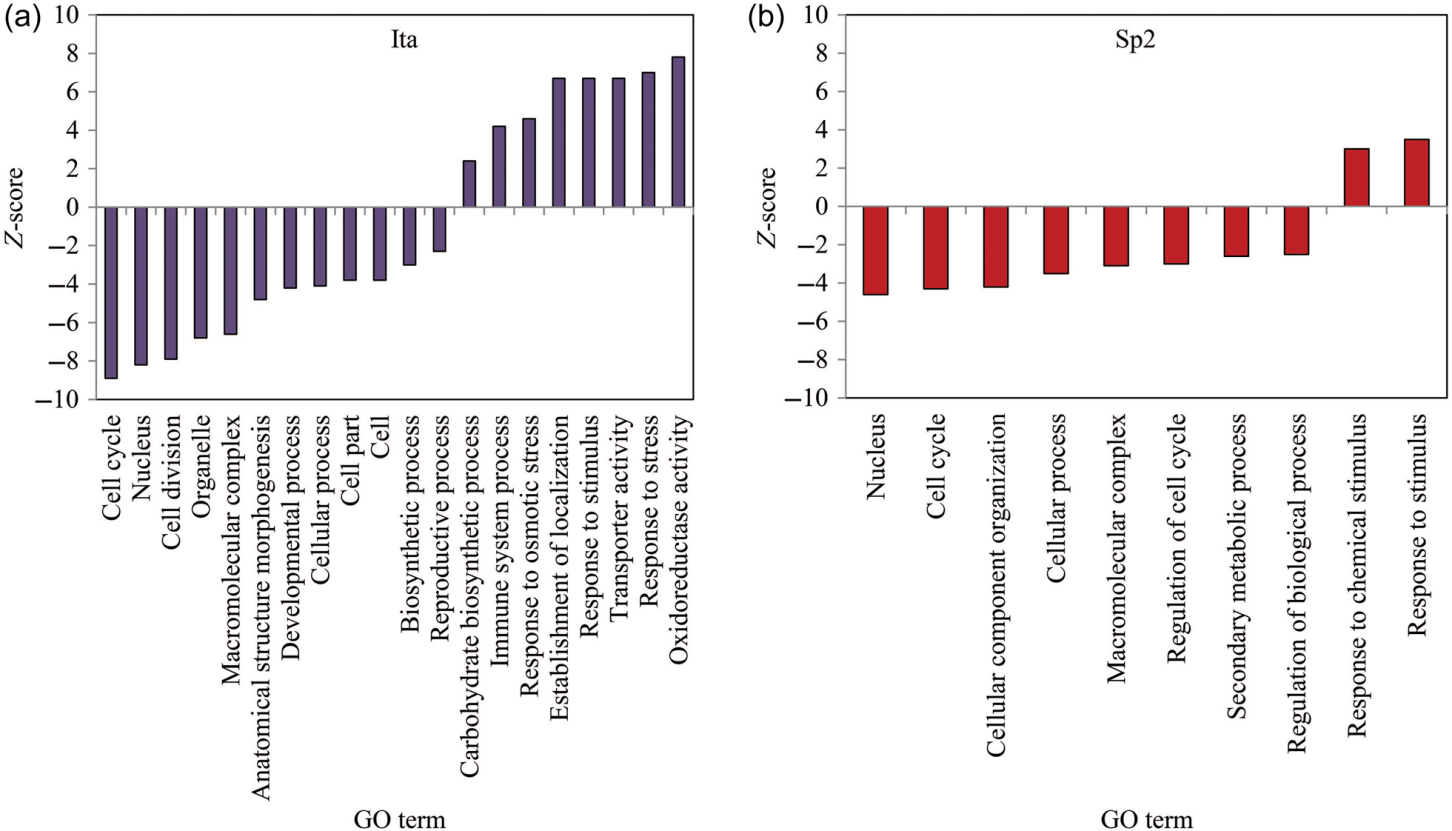
*Z*-score values of the main groups for Sp2 and Ita genotype transcripts in response to drought using the PAGE analysis from AgriGO (Du et al. 2010). Full analysis is in Table S9 available as Supplementary Data at *Tree Physiology* Online.

A combination of pathway analysis from MapMan and PAGE analysis from AgriGO allowed functional enrichments to be identified (Table 2, Figure 7). Only three BINs were significant in the MapMan analysis for the Spanish genotype in response to drought (Table 2, see Table S7 available as Supplementary Data at *Tree Physiology* Online for full details): cell (*P* = 0.0000003), secondary metabolism (*P* = 0.01) and transport (*P* = 0.000062). The Italian genotype had 24 MapMan BINs that were significant (*P* < 0.05) including DNA, RNA, cell, stress, transport, hormone metabolism and signalling (Table 2, see Table S7 available as Supplementary Data at *Tree Physiology* Online for full details).

**Table 2.** Description of the significant BINs from the microarray transcripts list in response to drought for the Spanish and the Italian genotypes. The probability (*P*-value) was calculated using a Wilcoxon Rank Sum test with a Benjamini–Hochberg correction in MapMan (Thimm et al. 2004). Examples of significant transcripts are given for several significant BINs with the probe set ID, Poplar gene model, a brief description and log_2_. The complete list is in Table S7 available as Supplementary Data at *Tree Physiology* Online.

Genotype	BIN code	BIN name	Probe set ID	Poplar gene model	*Arabidopsis* gene model	Brief description	Log_2_ (FC)
Ita	28	DNA (128 probes, *P* = 7.87E-15)					
	28.1	DNA synthesis/chromatin structure	ptpaffx.200289.1.s1_at	Potri.001G074000	AT5G44635.1	Minichromosome maintenance (MCM2/3/5) family protein	−4.97
	28.1.3	DNA synthesis/chromatin structure histone	ptp.4194.1.s1_x_at	Potri.017G123700	AT3G45980.1	Histone superfamily protein	−1.62
	28.2	DNA repair	ptp.1405.1.s1_at	Potri.014G128500	AT2G47590.1	Photolyase/blue-light receptor 2	−1.48
Ita	31	Cell (213 probes, *P* = 9.91E-18)					
	31.2	Cell division	ptpaffx.204723.1.s1_at	Potri.009G089200	AT3G19590.1	Transducin/WD40 repeat-like superfamily protein	−4.20
	31.3	Cell cycle	ptpaffx.200879.1.s1_at	Potri.001G272000	AT2G26760.1	Cyclin B1;4	−4.69
	31.4	Cell vesicle transport	ptpaffx.2864.2.s1_at	Potri.003G177700	AT1G04760.1	Vesicle-associated membrane protein 726	1.80
Ita	20	Stress (148 probes, *P* = 0.004)					
	20.1	Stress biotic	ptp.6055.1.s1_at	Potri.007G043500	AT4G37000.1	Accelerated cell death 2 (ACD2)	1.65
	20.2.1	Stress abiotic heat	ptpaffx.210289.1.s1_at	Potri.012G017600	AT5G42020.1	Heat shock protein 70 (Hsp 70) family protein	1.38
	20.2.3	Stress abiotic drought/salt	ptpaffx.208807.1.s1_x_at	Potri.010G094100	AT1G26850.1	*S*-adenosyl-l-methionine-dependent methyltransferases superfamily protein	−1.83
Ita	27	RNA (559 probes, *P* = 0.04)					
	27.3.3	RNA regulation of transcription AP2/EREBP, APETALA2/ethylene-responsive element binding protein family	ptpaffx.211416.1.s1_at	Potri.014G008100	AT4G37750.1	ANT, integrase-type DNA-binding superfamily protein	−3.23
	27.3.6	RNA regulation of transcription basic helix–loop–helix (bHLH)	ptpaffx.210224.1.s1_at	Potri.012G031800	AT5G53210.1	SPCH, bHLH DNA-binding superfamily protein	−2.70
	27.3.22	RNA regulation of transcription HB transcription factor family	ptpaffx.38907.1.s1_at	Potri.011G098300	AT2G34710.1	PHB, Homeobox-leucine zipper family protein/lipid-binding START domain-containing protein	−2.21
	27.3.25	RNA regulation of transcription MYB domain transcription factor family	ptpaffx.212699.1.s1_at	Potri.015G041100	AT1G22640.1	myb domain protein 3	−1.61
	27.3.32	RNA regulation of transcription WRKY domain transcription factor family	ptpaffx.203170.1.s1_at	Potri.003G111900	AT2G30590.1	WRKY DNA-binding protein 21	2.06
	27.3.50	RNA regulation of transcription General Transcription	ptpaffx.200328.1.s1_s_at	Potri.001G082700	AT4G24150.1	Growth-regulating factor 8	−2.42
Sp2	31	Cell (57 probes, *P* = 3.34E-7)					
	31.1	Cell organization	ptpaffx.148282.1.s1_s_at	Potri.002G111900	AT1G50010.1	Tubulin α-2 chain	−1.52
	31.2	Cell division	ptpaffx.212842.1.s1_at	Potri.015G090600	AT3G25100.1	Cell division cycle 45	−1.41
	31.3	Cell cycle	ptpaffx.63679.1.a1_s_at	Potri.005G181400	AT1G44110.1	Cyclin A1;1	−2.56

**Figure 8.**
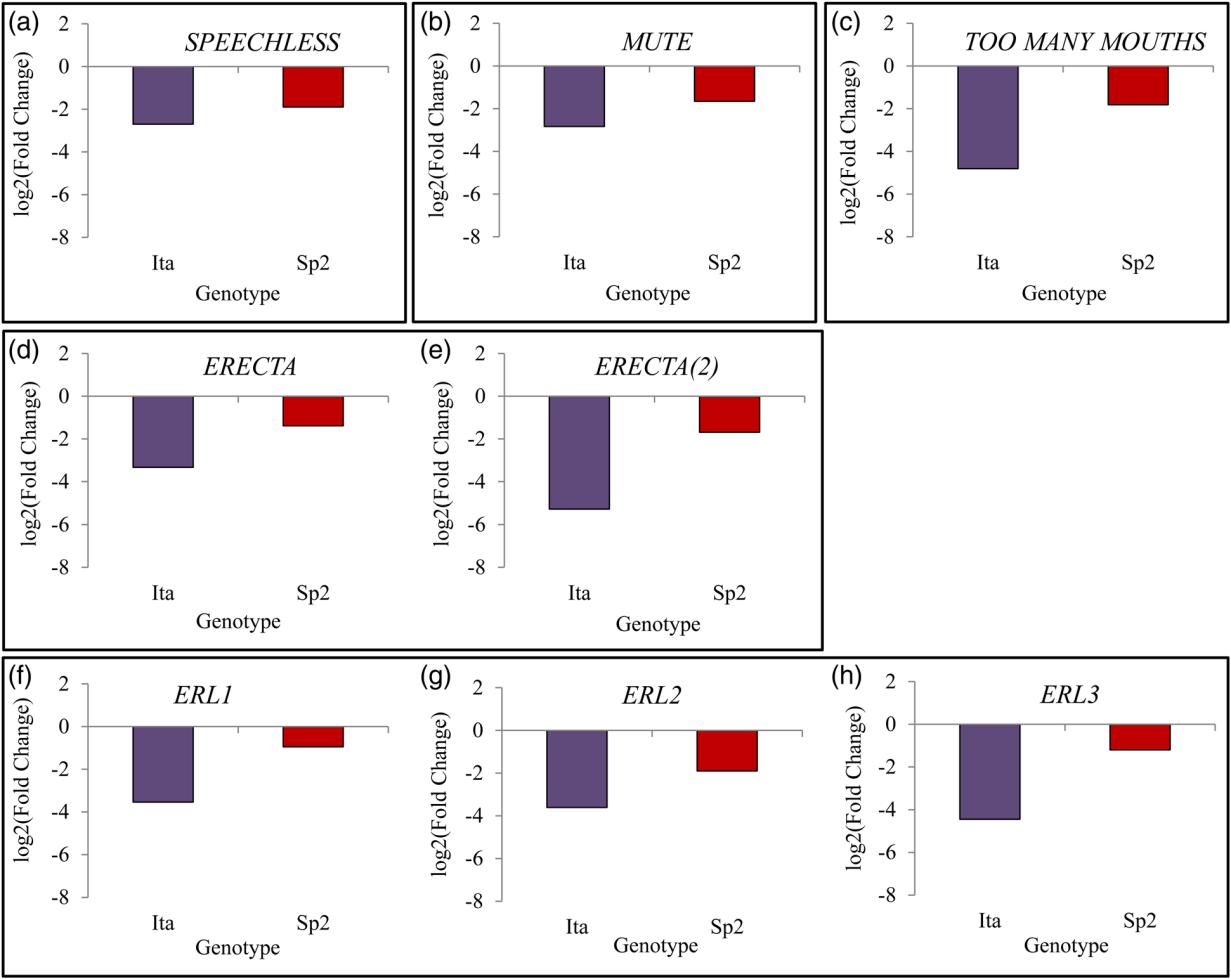
Gene expression changes for Sp2 and Ita in response to water deficit for stomatal patterning candidate genes: *ERECTA*, *ERL1*, *ERL2*, *ERL3*, *TMM*, *SPCH* and *MUTE*. Values are in log_2_.

Parametric analysis of gene set enrichment analysis of drought-responsive genes confirmed the results from MapMan and allowed 453 and 115 significantly enriched GO terms to be highlighted for Ita and Sp2, respectively (see Table S8 available as Supplementary Data at *Tree Physiology* Online). Eighty-three GO terms were commonly enriched for both genotypes. Among the 31 common down-regulated biological processes (see Table S8 available as Supplementary Data at *Tree Physiology* Online), 50% were related to cell division (e.g., ‘mitosis’, ‘DNA metabolic process’, ‘chromosome organization’ and ‘cell cycle’). Other negatively regulated processes were also found such as ‘regulation of gene expression’ and ‘secondary metabolic process’. Additionally, GO analysis revealed enrichment of up-regulated biological processes related to transport (GO:0006810, GO:0006812 and GO:0006811), response to stress and stimuli (GO:0006950, GO:0042221, GO:0050896 and GO:0009628) and carbohydrate catabolism (GO:0016052, GO:0019320, GO:0006007, GO:0046365 and GO:0006090) for both Ita and Sp2 (Figure 7, see Table S8 available as Supplementary Data at *Tree Physiology* Online). For the Spanish genotype only, functional enrichment was detected for repressed processes such as phenylpropanoid and flavonoid biosynthesis, and for induced ones involved in nucleotide and lipid metabolisms. Among the 247 biological processes enriched specifically for Ita, 135 are up-regulated including GO terms assigned to response to hormone (ABA, auxin, cytokinin, salicylic acid and jasmonate), response to abiotic and biotic stress (e.g., ‘response to water deprivation’, ‘response to osmotic stress’, ‘response to oxidative stress’ and ‘response to biotic stimulus’), metabolism and catabolism of amino acid, and to transport (ion, carbohydrate peptide, etc.). Finally, 112 down-regulated biological processes were found to be enriched for Ita only and are predominantly related to growth, development, cell division and morphogenesis. Among these down-regulated developmental processes, of particular interest were ‘stomatal complex development’ and its parent term ‘organ development’, which encompassed drought-responsive genes. Among drought-responsive genes, of particular interest were those related to stomatal development and patterning (Figure 8) and leaf development (Table 3), since these showed marked differences between genotypes in response to drought. In Sp2, only four genes were significantly down-regulated in response to drought: two *ERECTA* genes (*ERECTA*), one *Erecta-like* coding gene (*ERL2*) and *MUTE*, an orthologue of *SPEECHLESS*, which did not lead to a functional enrichment. In contrast, eight transcripts determining stomatal patterning were down-regulated in Ita in response to drought, including two *SPEECHLESS* orthologues (*SPEECHLESS* and *MUTE*), two (*ERECTA*) coding genes (*ERECTA*), three *Erecta-like* coding genes (*ERL1*, *ERL2* and *ERL3*) and *TOO MANY MOUTHS* (*TMM*). Transcripts controlling the activity of the shoot apical meristem and leaf development were also down-regulated in the Italian genotype in response to drought (Table 3), such as five close homologues of *ASYMMETRIC LEAVES* coding genes (*AS1*: *Potri.017G112300*, *Potri.006G085900*, *Potri.004G102600* and *AS2*: *Potri. 010G177100*, *Potri.008G079800*), six homologues of *PHABULOSA* (*PHB*), *CLAVATA1* (*CLV1*) and five homologues of *AINTGUMENTA* (*ANT*). Two of the same homologues of *AS1* and *AS2* were down-regulated in Sp2, as well as in one of the *PHB* homologues, but in general, as for stomatal patterning transcripts, there were far fewer changes in Sp2 then in Ita for leaf development transcript response to drought.

**Table 3.** Candidate genes involved in leaf development differentially expressed under drought in the Italian (Ita) and Spanish (Sp2) genotypes. Details include the name of the gene and probe set ID, the poplar (v3.0) and *Arabidopsis* gene models, the log_2_ expression ratio for each genotype (in bold if *P* < 0.05) and a brief description of its function.

Name	Probe set ID	Poplar gene model (v3.0)	*Arabidopsis* gene model	Ita log_2_(FC)	Sp2 log_2_(FC)	Description
*AS1*	PtpAffx.163978.1.S1_at	Potri.004G102600	AT2G37630.1	**−2.70**	−1.87	Involved in specification of the leaf proximodistal axis
*AS1*	PtpAffx.2947.1.S1_at	Potri.017G112300	AT2G37630.1	**−2.92**	−0.24	Involved in specification of the leaf proximodistal axis
*AS1*	PtpAffx.2947.2.A1_at			**−1.66**	−0.31	
*AS1*	Ptp.4356.1.S1_at	Potri.006G085900	AT2G37630.1	**−4.93**	−0.92	Involved in specification of the leaf proximodistal axis
*AS2*	PtpAffx.207814.1.S1_at	Potri.008G079800	AT1G65620.1	**−2.91**	−2.20	Required for formation of a symmetric flat leaf lamina
*AS2*	PtpAffx.209221.1.S1_at	Potri.010G177100	AT1G65620.1	**−1.82**	−1.23	Required for formation of a symmetric flat leaf lamina
*AS2*	PtpAffx.44821.1.A1_s_at			**−2.07**	−1.04	
*CLV1*	PtpAffx.201597.1.S1_at	Potri.002G019900	AT1G75820.1	**−2.06**	−0.52	Controls shoot and floral meristem size
*PHB*	Ptp.548.1.S1_at	Potri.001G372300	AT2G34710.1	**−3.29**	−0.46	Controls adaxial–abaxial patterning
*PHB*	Ptp.548.1.S1_x_at			**−3.14**	−0.37	
*PHB*	PtpAffx.38907.1.S1_at	Potri.011G098300	AT2G34710.1	**−2.21**	−0.79	Controls adaxial–abaxial patterning
*ANT*	PtpAffx.1799.1.A1_at	Potri.014G008100	AT4G37750.1	**−4.26**	−1.19	Required for control of cell proliferation
*ANT*	PtpAffx.211416.1.S1_at			**−3.23**	−0.77	
*ANT*	PtpAffx.147010.1.A1_at	Potri.002G114800	AT4G37750.1	**−1.41**	−0.80	Required for control of cell proliferation
*ANT*	PtpAffx.34524.3.A1_a_at	Potri.005G148400	AT4G37750.1	**−4.35**	−2.21	Required for control of cell proliferation

#### Variation under well-watered conditions

To elucidate constitutive differences in gene expression between the Spanish and Italian genotypes that are present in well-watered conditions, a comparison was also completed for the control data (see Table S6 available as Supplementary Data at *Tree Physiology* Online). Two hundred and fifty-two up-regulated and 284 down-regulated transcripts were identified in Sp2 compared with Ita (Figure 6c). The AgriGO analysis showed enriched GO terms differentially expressed between Sp2 and Ita in well-watered conditions, and these were generally related to secondary metabolism. Also up-regulated in the Spanish genotype were *ERD1* (*EARLY RESPONSE TO DEHYDRATION 1*) and *RD21* (*RESPONSIVE TO DEHYDRATION 21*).

#### Real-time qPCR

Microarray results were validated by real-time qPCR on four candidate genes selected after microarray analysis. Gene expression was quantified for additional genotypes that were not included in the microarray experiment: Fr1 from France and NL from the Netherlands (see Figure S3 available as Supplementary Data at *Tree Physiology* Online). Real-time qPCR values were expressed in response to drought for each genotype. *SPEECHLESS* expression ratios were lower in response to drought in both Ita and Sp2, although this response was greater in Ita (*F*_3,32_ = 9.311, *P* < 0.001, see Figure S3 available as Supplementary Data at *Tree Physiology* Online). The expression ratios of *ERECTA* were reduced in response to drought with no significant difference between genotypes (*F*_3,32_ = 0.845, *P* = 0.48, see Figure S3 available as Supplementary Data at *Tree Physiology* Online).

## Discussion

Our analysis has revealed significant natural variation between populations of black poplar originating from contrasting climatic conditions within Europe. By combining a common garden approach with manipulative experiments and genome-wide gene expression, this study provides considerable insight into the intraspecific variation in drought tolerance for this important keystone riparian tree species. We have identified transcriptome and trait differences that suggest important adaptive mechanisms that exist within the species.

From results at a single site in northern Europe under well-watered conditions, leaf, cell and stem size traits differed among genotypes of *P. nigra* (Figure 1), we hypothesize that Spanish and southern French genotypes have smaller leaves as an adaptation to drought developed in their native environment. Similar observations have been drawn for two other genotypes of *P. nigra* from contrasting northern and southern (water limited) environments in Italy (Regier et al. 2009, Cocozza et al. 2010).

For the population of *P. nigra*, genotypic variation was clear in Δ^13^C and varied with site of origin (Figure 1). Wood Δ^13^C was lower in populations from the north and east of Europe, such as The Netherlands, Germany and northern Italy, and this indicates higher WUE. However, these trees were collected from wet environments in Europe, comparable to the conditions in the common garden, suggesting that they are particularly well adapted to the Belgian climate. On the other hand, and perhaps counter-intuitively, Spanish and southern French populations had the highest Δ^13^C, suggesting a lower WUE and poor control of water loss without a reduction in photosynthesis or lowered photosynthetic rates but with unchanged *g*_s_. In contrast to Δ^13^C, no significant differences were observed between populations in SI (a measure of stomatal patterning), although there was a trend of increased stomatal numbers in northern and eastern genotypes. Given the potential for stomatal patterning and related genes to affect *g*_s_ and thus WUE (Woodward et al. 2002, Masle et al. 2005, Roussel et al. 2009), this lack of significance was surprising. Any adaptation to water deficit by the small-leaf morphotypes, characteristic of Spanish trees, is likely, however, to involve additional physiological pathways that are distinct from those controlling stomatal development. It is also possible that stomatal patterning is phenotypically plastic, with differential stomatal patterning occurring in leaves in response to water deficit, and our data for gene expression from the drought experiment would support this contention.

Contrasting genotypes were identified from the moderate drought experiment on genotypes from four locations (Figure 3a), with different adaptive mechanisms apparent for response to drought stress. The ‘north eastern’ genotype is characteristic of the northern Italian and Netherlands genotypes, originating from areas of high precipitation, where tree productivity and leaf area development are generally high but where height growth and new leaf formation decreased dramatically following the onset of drought. In contrast, a ‘southern’ genotype, from a region of low precipitation, is characterized by the Spanish and southerly French populations. Slow-growing with small leaves, these genotypes responded to drought with rapid stomatal closure, with the maintenance of leaf expansion (for Sp2) and formation (at least in the extreme example of Sp2), but with some leaf loss. Rapid stomatal closure only 5 days after drought in French and Spanish genotypes supports the idea that variation in stomatal behaviour can exist within species, as was shown by Sparks and Black (1999) in four populations of *P. trichocarpa* originating from contrasting environments.

Stomatal closure is a biological process to avoid water loss in the event of drought stress but can have other physiological consequences as it can inhibit photosynthesis (Cornic 2000). There is a trend in our results, which indicates that Sp2 closed stomata more in response to drought when compared with Ita (5DAD and 15DAD) and this correlated with reduced Δ^13^C under drought, suggesting an increase in WUE in droughted conditions. In a study of δ^13^C in beech planted in different sites throughout Europe, the highest values (thus the lowest values of Δ^13^C) were observed in the most southern location in France (Keitel et al. 2006). Monclus et al. (2005) studied different genotypes of *Populus* (tolerant and non-tolerant to drought) and showed that the drought-tolerant trees tended to decrease in Δ^13^C, but the inverse was observed for the non-tolerant genotypes.

Genotypes from Spain and Italy were selected for gene expression analyses because their sites of origin differed markedly in rainfall but not temperature, and thus likely represented contrasting strategies for response to soil water deficits. Given the controlled application of water deficit, with controlled constant temperature in this experiment, it was surprising to see that gene expression changes differed so markedly between the two genotypes, with more than eight times the number of differentially expressed genes observed in the Ita compared with the Sp2 genotype. It is remarkable that only 901 transcripts were commonly expressed in response to drought for both genotypes, considering >8000 changes in gene expression were observed in total. This result strongly suggests that the Spanish and Italian genotypes are differentially adapted to drought stress and that this involves considerable plasticity in gene expression—manifested in contrasting phenotypic acclimation to the imposed stress. This result is similar to that from Mediterranean species, which has suggested that phenotypic plasticity is lower in plants from low resource environments as part of a conservative resource-use strategy (Valladares et al. 2000). These contrasting patterns of gene expression in *P. nigra*, and their associated phenotypes, provide important clues to aid our understanding of adaptation. This will help to ensure the availability of a resilient gene pool as drought stress increases across Europe, which is a valuable resource for future management and conservation of black poplar.

**Figure 9.**
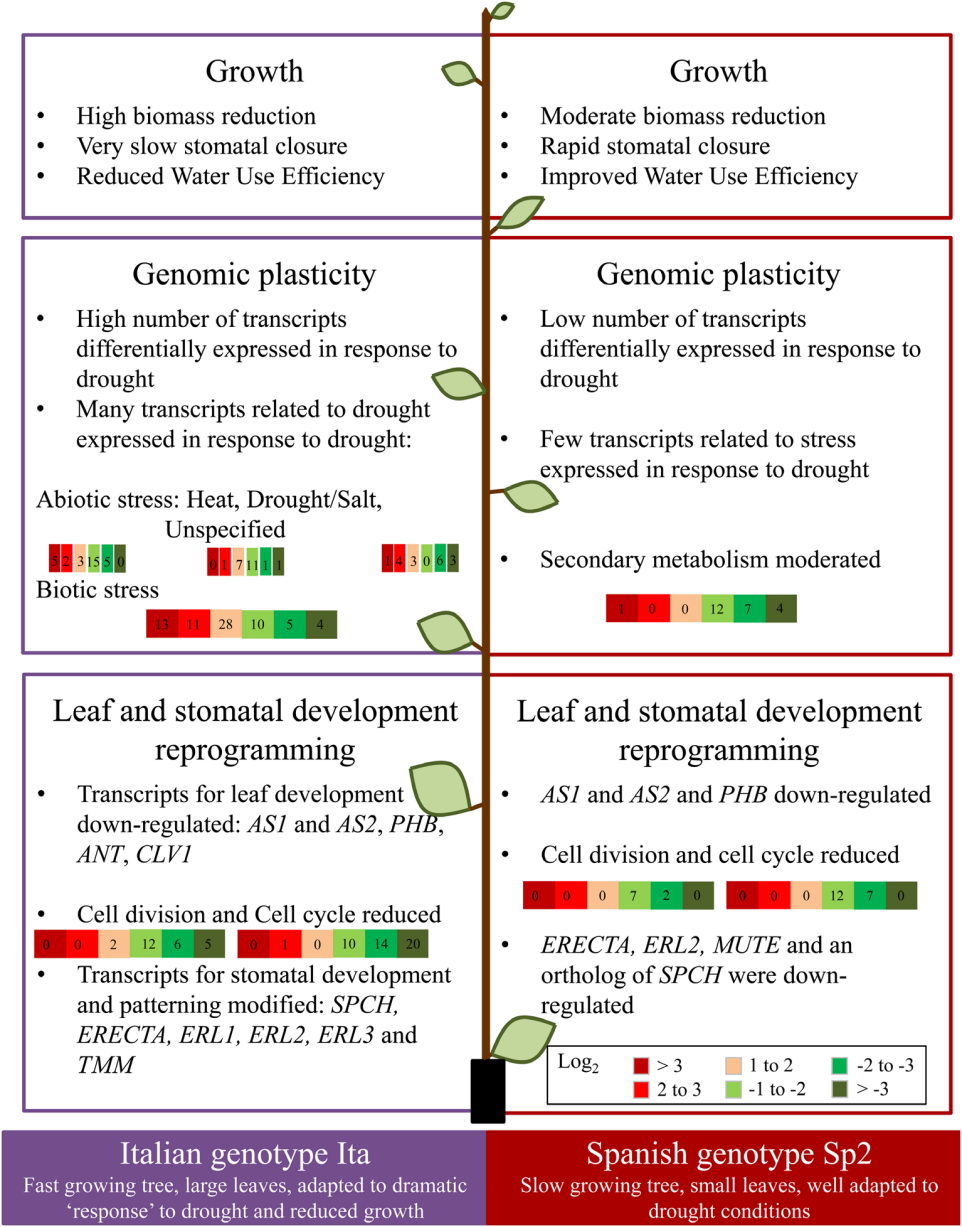
Summary of the response to drought in two genotypes of *P. nigra*.

A larger number of GO groups related to ‘response to stimulus’ were significantly enriched in Ita compared with in Sp2, suggesting a highly water stress-responsive gene expression pattern in the Ita trees (Figure 9). Similar conclusions were drawn for salt-stressed (Walia et al. 2005, 2007) and drought-stressed (Degenkolbe et al. 2009) rice genotypes. When comparing two genotypes of potatoes, Schafleitner et al. (2007) observed only 186 up-regulated and 77 down-regulated genes in common, while 1713 genes were expressed in total in response to drought.

There are two important phenotypic traits that underpin drought tolerance and appear to be key to understanding genomic plasticity and adaptation in these contrasting genotypes of black poplar. These traits are linked to leaf development and stomatal patterning and contribute to drought tolerance. Leaf size determines leaf and canopy transpiration (Radin et al. 1994, Levi et al. 2009, Ashraf 2010) and is also tightly related to yield, an important trait linked to fitness (Rae et al. 2004, Monclus et al. 2005). Leaf production and leaf loss represent important adaptive mechanisms enabling long-lived trees to moderate the amount of transpiring leaf surface area. Furthermore, stomatal aperture and stomatal number both contribute to the control of transpiration, leaf-level WUE and drought tolerance (Nilson and Assmann 2007). While both genotypes showed reduced leaf expansion in response to drought, for the Ita genotype, this reduction was dramatic (<50%), while for Sp2, it was moderate (<20%). Changes in gene expression concur with these different developmental responses to drought. Down-regulation of genes involved in balance between shoot apical meristem activity and the initiation, and development of leaf primordia such as *ANT*, *PHB*, *AS1*, *AS2* and *CLV1* concur with these developmental responses to drought in the Italian genotype, while only *AS1* and *AS2* were down-regulated in Sp2 (Table 3). Together with the drastic down-regulation of processes related to growth, development and cell division revealed by PAGE analysis, these results suggest that cell proliferation, leaf expansion and leaf size would be reduced for the Ita genotype in response to drought, while the Sp2 genotype would be less affected. Ita expression is thus more concentrated in reacting to stress rather than maintaining leaf development (Figure 9), and this is supported by a drastic down-regulation of processes linked to cell division revealed by GO analysis.

Similarly, striking differences in genes controlling stomatal initiation and number were observed in response to drought for Spanish and Italian genotypes. Stomata regulate CO_2_ and water-vapour exchange between leaves and the atmosphere (MacAlister et al. 2007) and prevent water loss through partial stomatal closure. Although the genetic control of stomatal initiation and patterning is now well documented (Barton 2007, Gray 2007, Casson and Hetherington 2010, Torii 2015), less is known about how the environment interacts with the control of stomatal patterning, although genes regulating the development of stomata have also been discovered in response to light (Casson et al. 2009), CO_2_ (Gray et al. 2000, Hu et al. 2010) and drought (Masle et al. 2005). Unfortunately stomatal patterning was not measured here, but our ongoing research suggests that patterning differs depending on genotype (H. Smith, unpublished data).

Several stomatal patterning genes that negatively regulate stomatal number were down-regulated in response to drought for the Italian genotype including *TMM* (*TOO MANY MOUTHS*), *ERECTA* and *ERL1* (*ERECTA-LIKE 1*, Figure 8). In particular, there is strong evidence that increased transcript abundance in *ERECTA* is linked to declining stomatal numbers, and that *ERECTA* acts to regulate the initial decision of cells to enter the stomatal developmental pathway (Shpak et al. 2007). Two positive regulators of stomatal development were also down-regulated—*SPEECHLESS* and *MUTE*. Overall, the down-regulation of *ERECTA*, *ERL1* and *TMM* in the Italian genotype suggests that the formation of stomata was stimulated in response to drought. Few changes in gene expression for genes that regulate stomatal numbers were apparent for the Spanish ecotype; only the *ERECTA* gene showed any response to drought, and this could still be significant. The stomatal patterning phenotype remains to be tested in these *P. nigra* trees.

Although our prediction is for increased stomatal numbers in response to drought for Ita, this has not yet been tested but is the subject of future research alongside RNA-Seq analysis of guard cell and epidermal gene expression. The stimulation of stomatal initiation in response to drought is somewhat counter-intuitive, and recent reports for *P. balsamifera* showed reduced stomatal numbers following drought treatment (Hamanishi et al. 2012). These authors also assessed expression of several stomatal patterning genes and differences between two commercial genotypes were apparent, although they were often inconsistent across several sampling times. Nevertheless, they reinforce the concept that the regulation of stomatal numbers varies intraspecifically and may be an important control point to elucidate differences in adaptation to drought in the genus *Populus* (Roussel et al. 2009).

In summary, we have identified significant differences in response to drought for black poplar genotypes collected from dry and wet environments across Europe. ‘Southern’ Spanish trees are well adapted to slow growth in droughted conditions, producing small leaves and partially closed stomata, with a higher intrinsic WUE, while Italian and ‘north eastern’ trees demonstrate a dramatic response to drought with reduced growth and increased stomatal formation. We hypothesize, therefore, that each of these strategies may be of value, depending on the likely frequency and duration of drought in a particular environment. Importantly here, we have identified a suite of genes that will be the focus of our future research using reverse genetic approaches and testing material in the field in contrasting drought environments. Thus, screening for functional genomic and genetic variation in genotypes from diverse geographic locations under drought stress is a powerful strategy to inform the conservation and management of germplasm resources in a future, changing climate and should be exploited more widely in these difficult-to-study, long-lived but critical plants that contribute to timber, fuel, fibre and ecosystem service provision on a global scale.

## Supplementary data

Supplementary data for this article are available at *Tree Physiology* Online.

## Conflict of interest

None declared.

## Funding

This research was supported by the European Commission through the Directorate General Research within the Fifth Framework for Research – Quality of Life and Management of the Living Resources Programme, contact No. QLK5-CT-2002-00953 (POPYOMICs), within the Sixth Framework for Research as part of the Network of Excellence EVOLTREE, contract No. 016322 – Sustainable development, Global Change and Ecosystems Programme and through the Seventh Framework for Research, Food Agriculture and Fisheries, Biotechnology, within the project ENERGYPOPLAR, contract No. FP7-211917 and WATBIO, contract No. FP7-311929. H.T. was supported by a PhD studentship from NERC. Funding to pay the Open Access publication charges for this article was provided by RCUK.

## Supplementary Material

Supplementary Data
