# Supplementary material for: Adaptive mechanisms and genomic plasticity for drought tolerance identified in European black poplar (Populus nigra L.)
Source: Tree Physiol. 2016 Aug 1;36(7):909–28. doi: 10.1093/treephys/tpw017 (PMC4969554; doi:10.1093/treephys/tpw017)
Supplement: Supplementary Data [file supp_36_7_909__index.html]

Adaptive mechanisms and genomic plasticity for drought tolerance identified in European black poplar (Populus nigra L.) — Supplementary Data 

# Adaptive mechanisms and genomic plasticity for drought tolerance identified in European black poplar (*Populus nigra* L.)

## Supplementary Data

Supplementary Data

- Supplementary Data - Docx file
- Supplementary Figure 1 - jpg file
- Supplementary Figure 2 - JPG file
- Supplementary Figure 3 - JPG file
- Supplementary Table\_S1,S2,S3,S5 - docx file
- Supplementary Table\_S4 - xlsx file
- Supplementary Table\_S6 - xlsx file
- Supplementary Table\_S7 - xls file
- Supplementary Table\_S8 - xlsx file
