# Supplementary material for: Adaptive mechanisms and genomic plasticity for drought tolerance identified in European black poplar (Populus nigra L.)
Source: Tree Physiol. 2016 Aug 1;36(7):909–28. doi: 10.1093/treephys/tpw017 (PMC4969554; doi:10.1093/treephys/tpw017)
Supplement: Supplementary Data [file supp_tpw017_tpw017supp.docx]

# Supplementary documents:

Table S1: Details of the populations of *Populus nigra*, their location and climates. Temperature and precipitation data were collected from the website <http://www.worldclim.org/>. Range of temperatures and precipitations are given for the population collected at different locations (e.g. along a river system).

Table S2: Provenance of the six *P. nigra* genotypes used in the drought experiment.

Table S3: Forward and Reverse primers for each candidate gene (5’ to 3’).

Table S4: Climatic data from the region of origin and phenotypic correlation among traits measured in a common garden study of *P. nigra* from natural populations across western Europe. Correlation coefficients were estimated as Spearman’s rho, with p-values corrected for multiple comparisons using the sequential Bonferroni (asterisks).

Table S5: Summary of the measurements for each genotype under well-watered (control) and drought treatments. Average ± standard error.

Table S6: Normalised microarray expression matrix using the complete list of transcripts.

Table S7: Complete Mapman analysis. Description of the significant bins from the microarray transcripts list in response to drought for the Spanish and the Italian genotypes. The probability (p-value) was calculated using a Wilcoxon Sum of Rank test with a Benjamini Hochberg correction in MapMan (Thimm *et al.*, 2004). Complete list of significant transcripts are given for each significant bin with the probe set ID, Poplar gene model, a brief description, and log_2_ expression ratio.

Table S8: Complete AgriGO analysis with all the significant GO groups per genotype, the description of the GO term, the number of transcripts, the Z-score and the False Discovery Rate (FDR).

Figure S1: Volumetric soil moisture at final day of harvest **(a)** and rate of soil drying ((final soil moisture – initial soil moisture)) x 100 **(b)**.

Figure S2: Volumetric soil moisture at DAD5 when stomatal conductance measurements were taken.

Figure S3: Expression ratio (log_2_) from real-time qPCR in response to drought per genotype for the candidate genes: *SPEECHLESS* **(a)**, *IP3* **(b)**, *ATHVA22A* **(c)** and *ERECTA* **(d)**. Same letter indicate no significant difference at 5% level, Student-Newman Keuls post-hoc testing. Each value with bars represents the average ± standard error.
