# Supplementary figures and images for: Adaptive mechanisms and genomic plasticity for drought tolerance identified in European black poplar (Populus nigra L.)
Source: Tree Physiol. 2016 Aug 1;36(7):909–28. doi: 10.1093/treephys/tpw017 (PMC4969554; doi:10.1093/treephys/tpw017)

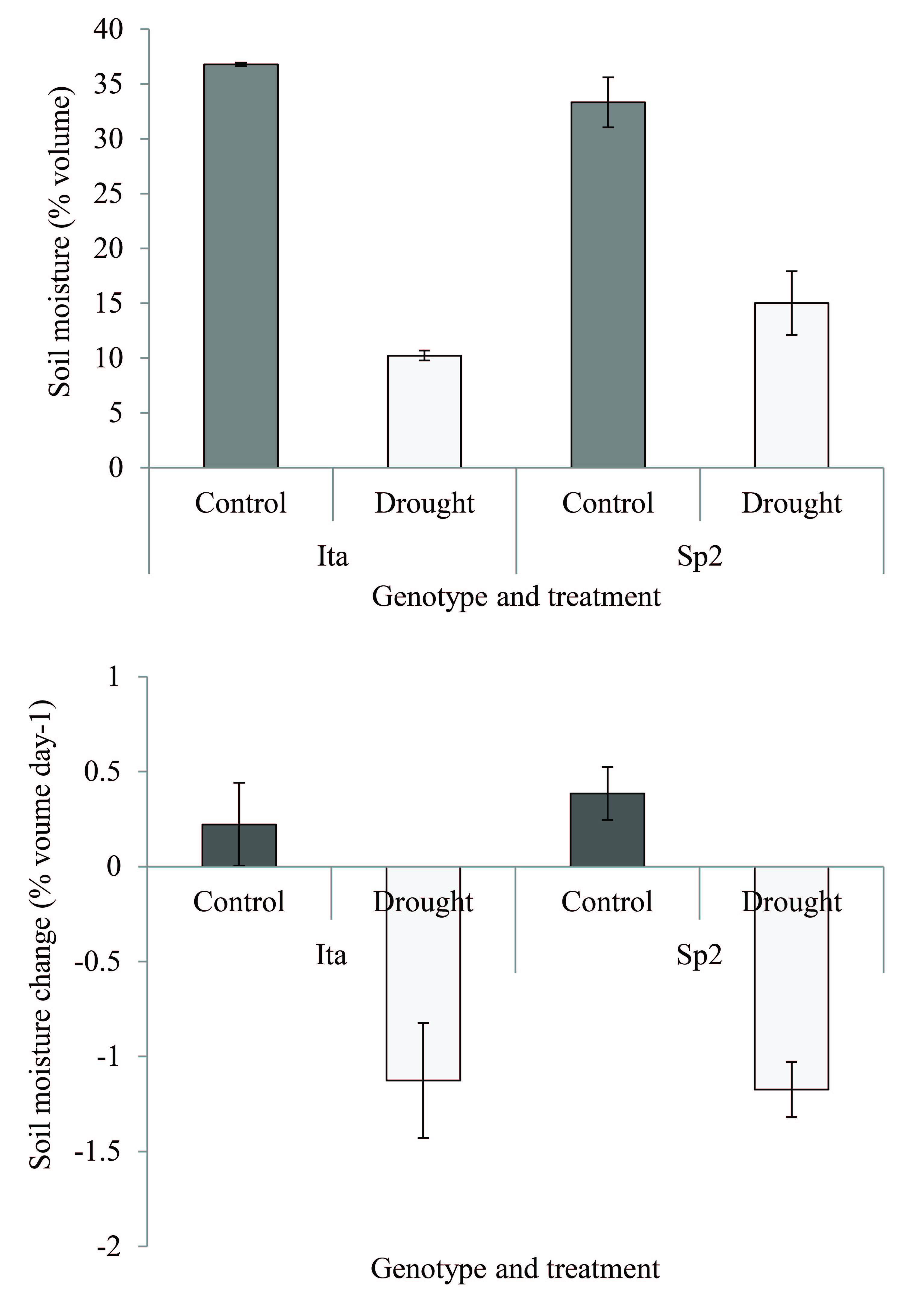

Supplement: Supplementary Data [file supp_tpw017_tpw017supp_fig1.jpg]

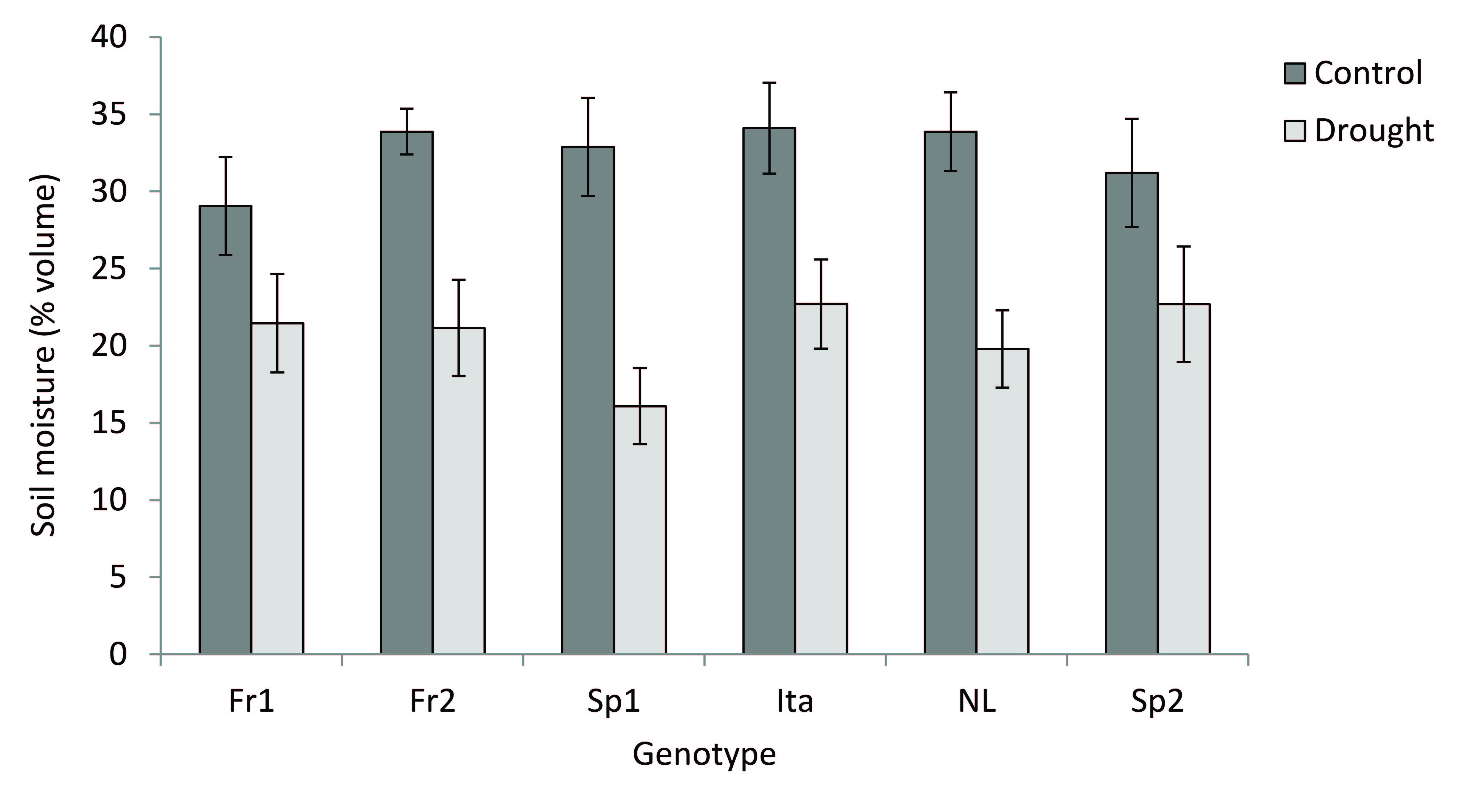

Supplement: Supplementary Data [file supp_tpw017_tpw017supp_fig2.jpg]

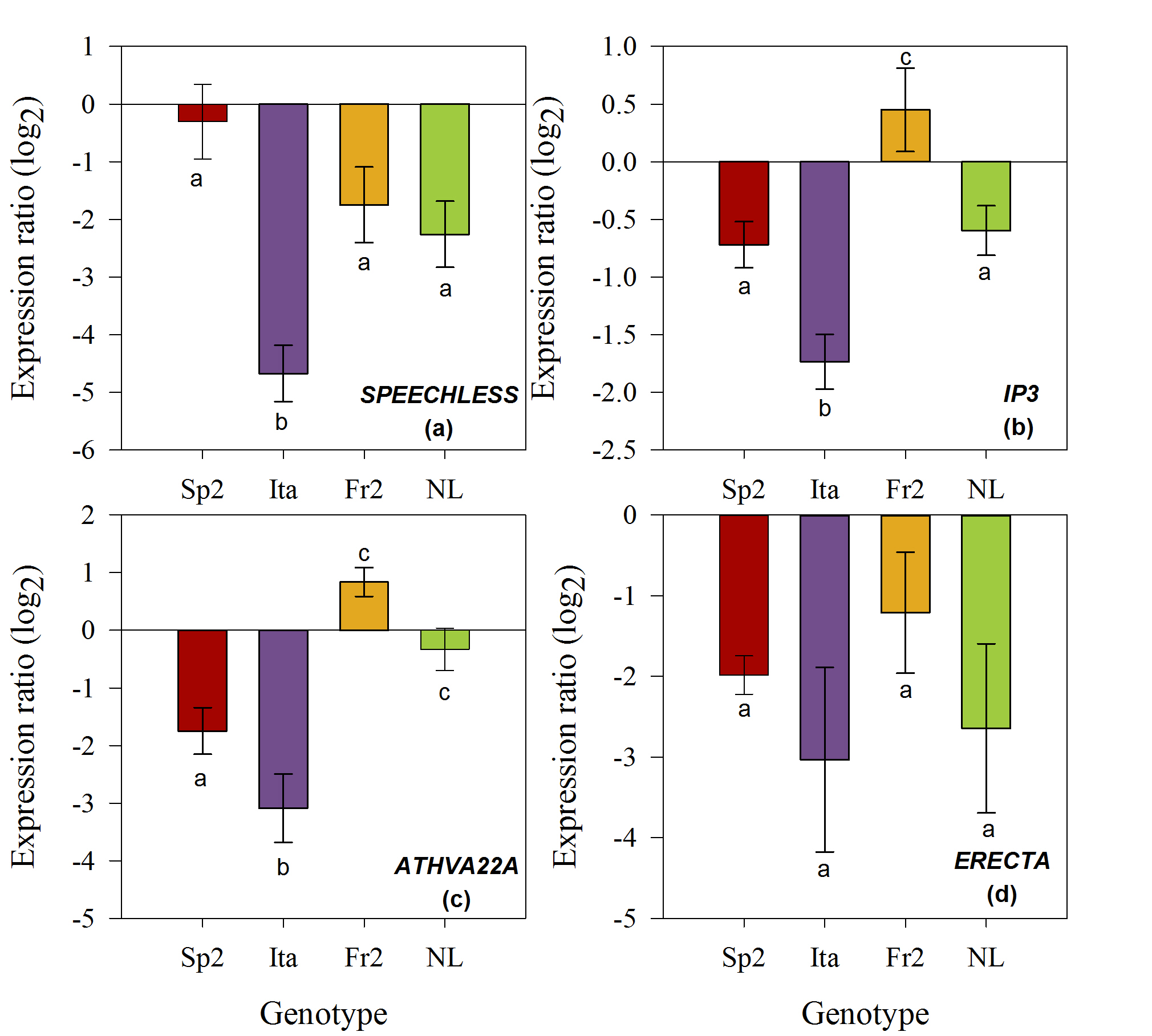

Supplement: Supplementary Data [file supp_tpw017_tpw017supp_fig3.jpg]
