# Supplementary material for: Adaptive mechanisms and genomic plasticity for drought tolerance identified in European black poplar (Populus nigra L.)
Source: Tree Physiol. 2016 Aug 1;36(7):909–28. doi: 10.1093/treephys/tpw017 (PMC4969554; doi:10.1093/treephys/tpw017)
Supplement: Supplementary Data [file supp_tpw017_tpw017supp_Table_S1S2S3S5.docx]

Table S1: Details of the populations of *Populus nigra*, their location and climates. Temperature and precipitation data were collected from the website <http://www.worldclim.org/>. Range of temperatures and precipitations are given for the population collected at different locations (e.g. along a river system).

| **Population name** | **Latitude** | **Longitude** | **Country** | **River system** | **Collection** | **Average annual temperature (°C)** | **Maximum temperature of warmest month (°C)** | **Minimum temperature of coolest month (°C)** | **Average annual precipitation (mm)** | **Precipitation of wettest month (mm)** | **Precipitation of driest month (mm)** |
| --- | --- | --- | --- | --- | --- | --- | --- | --- | --- | --- | --- |
|  |  |  |  |  |  |  |  |  |  |  |  |
| Loire Est | 47°28’N | 02°54’E | France | Loire | Along the river | 10.8 - 11.1 | 24.7 - 24.9 | -0.4 - 0 | 657 - 744 | 66 - 76 | 47 - 49 |
| Loire W | 47°28’N | 00°33’W | France | Loire | Along the river | 11.3 - 11.7 | 23.7 - 25 | 1 - 2.3 | 650 - 779 | 70 - 87 | 43 - 45 |
| Drôme1 | 44°41’N | 05°24’E | France | Drôme | Exact location | 10.3 | 25.7 | -2.4 | 890 | 83 | 48 |
| Drôme6 | 44°45’N | 04°55’E | France | Drôme | Exact location | 12.4 | 28.1 | 0 | 840 | 95 | 41 |
| Durance | 43°42’N | 05°22’E | France | Durance | Along the river | 12.2 - 13.6 | 26.7 - 28.7 | 0 - 1.1 | 639 - 732 | 84 - 90 | 21 - 28 |
| Ebro1 | 41°56’N | 01°23’W | Spain | Ebro | Exact location | 14.1 | 29.7 | 1.8 | 439 | 56 | 20 |
| Ebro2 | 41°35’N | 01°00’W | Spain | Ebro | Exact location | 13.7 | 29.5 | 1.3 | 365 | 53 | 17 |
| Rhine | 49°49’N | 08°30’E | Germany | Rhine | Exact location | 9.8 | 24.5 | -2 | 605 | 67 | 37 |
| Ticino W | 45°16’N | 08°59’E | Italy | Ticino | Exact location | 13 | 29 | -1 | 982 | 122 | 55 |
| Ticino Est | 45°12’N | 09°04’E | Italy | Ticino | Exact location | 13 | 29 | -0.9 | 966 | 121 | 55 |
| Netherlands | 52°02’N | 05°13’E | Netherlands | | Around the location | 8.6 - 10.2 | 20.6 - 21.8 | -1.3 - 1.3 | 731 - 1021 | 73 - 100 | 43 - 73 |

Table S2: Provenance of the six *P. nigra* genotypes used in the drought experiment

| Genotype | River population | Country | Number of replicates control | Number of replicates drought |
| --- | --- | --- | --- | --- |
| Sp1 | Ebro1 | Spain | 8 | 9 |
| Sp2 | Ebro2 | Spain | 5 | 8 |
| Fr1 | Drôme6 | France | 9 | 10 |
| Fr2 | Drôme6 | France | 10 | 10 |
| Ita | Ticino (left side) | Italy | 10 | 10 |
| NL | Netherlands | Netherlands | 10 | 10 |

Table S3: Forward and Reverse primers for each candidate genes (5’ to 3’)

| **Gene name** | **Forward primer (5’ to 3’)** | **Reverse primer (5’ to 3’)** |
| --- | --- | --- |
| *SPEECHLESS* | GCCCATTAGCCCAAGAAC | GGGCCTGAGAATTTAACC |
|  |  |  |
|  |  |  |
| *ERECTA* | GAATTGTCCCTCCATGAGC | TGCTCGATACTGCTCTGC |
|  |  |  |


Table S5: Summary of the measurements for each genotype under well-watered (control) and drought treatments. Average ± standard error

|  |  | Height growth (mm) | Stem diameter  growth (mm) | Number of leaves developed | Number of fallen leaves | Number of  branches developed | SLA (cm^2^/g) | g_s_ 5DAD  (µmol m^-2^ s^-1^) | g_s_ 15DAD  (µmol m^-2^ s^-1^) | Δ^13^C  (‰) |
| --- | --- | --- | --- | --- | --- | --- | --- | --- | --- | --- |
|  |  |  |  |  |  |  |  |  |  |  |
| Sp1 | control | 13.62±2.14 | 0.67±0.19 | 8.88±0.48 | 8.50±1.48 | 4.17±1.74 | 205.58±8.88 | 471.62±23.28 | 408.37±37.20 | 22.49±0.22 |
|  | drought | 6.00±0.80 | 0.31±0.13 | 6.33±0.41 | 13.00±3.07 | 2.50±1.70 | 181.74±5.29 | 297.18±81.61 | 110.54±14.36 | 19.83±0.51 |
| Sp2 | control | 8.8±2.41 | 0.67±0.15 | 6.8±1.02 | 12.20±2.06 | 2.00±4.00 | 229.09±22.97 | 600.8±83.53 | 388.60±90.65 | 24.36±0.90 |
|  | drought | 5.56±1.21 | 0.36±0.10 | 6.00±0.65 | 16.62±3.62 | 4.00±1.81 | 212.49±14.30 | 383.65±92.22 | 112.42±15.79 | 21.95±1.32 |
| Fr1 | control | 10.75±1.24 | 0.68±0.09 | 6.00±0.62 | 10.33±2.30 | 0.00±0.00 | 222.21±7.45 | 275±27.00 | 284.56±40.44 | 22.43±0.11 |
|  | drought | 7.28±1.48 | 0.20±0.08 | 4.50±0.53 | 26.50±6.62 | 0.44±0.58 | 205.01±10.73 | 166.95±69.92 | 99.19±32.61 | 19.90±0.40 |
| Fr2 | control | 8.35±1.03 | 1.04±0.20 | 5.40±0.56 | 6.40±1.57 | 3.87±4.16 | 185.41±5.62 | 378.30±49.51 | 266.43±40.00 | 21.26±0.11 |
|  | drought | 3.95±0.84 | 0.27±0.12 | 3.60±0.34 | 10.40±3.31 | 0.00±0.44 | 190.79±8.57 | 172.85±47.16 | 120.13±28.29 | 21.21±0.94 |
| Ita | control | 5.22±1.18 | 0.17±0.16 | 3.89±0.48 | 6.12±1.37 | 6.11±5.99 | 243.50±15.75 | 338.70±32.00 | 301±13.13 | 23.27±0.73 |
|  | drought | 0.70±0.97 | 0.25±0.26 | 2.62±0.37 | 6.62±1.57 | 0.00±0.00 | 251.75±15.38 | 350.22±20.54 | 164.09±40.19 | 24.62±0.81 |
| NL | control | 11.14±1.39 | 0.50±0.09 | 5.20±0.53 | 7.40±1.17 | 0.00±0.00 | 297.04±27.10 | 375.9±23.27 | 289.5±25.41 | 23.40±0.43 |
|  | drought | 6.17±1.57 | 0.30±0.15 | 3.67±0.53 | 8.67±1.05 | 0.00±0.29 | 251.97±17.89 | 311.31±58.22 | 121.33±31.71 | 22.70±0.99 |
